# The Strain-Encoded Relationship between PrP^Sc^ Replication, Stability and Processing in Neurons is Predictive of the Incubation Period of Disease

**DOI:** 10.1371/journal.ppat.1001317

**Published:** 2011-03-17

**Authors:** Jacob I. Ayers, Charles R. Schutt, Ronald A. Shikiya, Adriano Aguzzi, Anthony E. Kincaid, Jason C. Bartz

**Affiliations:** 1 Department of Medical Microbiology and Immunology, Creighton University, Omaha, Nebraska, United States of America; 2 Institute of Neuropathology, University Hospital Zurich, Zurich, Switzerland; 3 Department of Physical Therapy, Creighton University, Omaha, Nebraska, United States of America; University of Edinburgh, United Kingdom

## Abstract

Prion strains are characterized by differences in the outcome of disease, most notably incubation period and neuropathological features. While it is established that the disease specific isoform of the prion protein, PrP^Sc^, is an essential component of the infectious agent, the strain-specific relationship between PrP^Sc^ properties and the biological features of the resulting disease is not clear. To investigate this relationship, we examined the amplification efficiency and conformational stability of PrP^Sc^ from eight hamster-adapted prion strains and compared it to the resulting incubation period of disease and processing of PrP^Sc^ in neurons and glia. We found that short incubation period strains were characterized by more efficient PrP^Sc^ amplification and higher PrP^Sc^ conformational stabilities compared to long incubation period strains. In the CNS, the short incubation period strains were characterized by the accumulation of N-terminally truncated PrP^Sc^ in the soma of neurons, astrocytes and microglia in contrast to long incubation period strains where PrP^Sc^ did not accumulate to detectable levels in the soma of neurons but was detected in glia similar to short incubation period strains. These results are inconsistent with the hypothesis that a decrease in conformational stability results in a corresponding increase in replication efficiency and suggest that glia mediated neurodegeneration results in longer survival times compared to direct replication of PrP^Sc^ in neurons.

## Author Summary

Prion diseases are a group of infectious fatal neurodegenerative diseases that affect animals including humans. This unique infectious agent is the result of a post-translational conformational change of the normal form of the prion protein, PrP^C^, to an infectious form of the prion protein, PrP^Sc^. Different strains of the infectious agent result in characteristic incubation periods and neuropathological features within a single host species. These strain-specific differences in disease outcome are likely due to strain-specific conformations of PrP^Sc^, though the mechanisms by which different conformation can affect prion strain properties are not understood. The aim of this study was to investigate the relationship between the biochemical properties of PrP^Sc^ to the corresponding neuropathological characteristics of eight hamster-adapted prion strains. Our findings indicate that PrP^Sc^ from short incubation period strains were more efficiently replicated, had a more stable conformation, and were observed to be more resistant to clearance from the soma of neurons compared to prion strains with a relatively long incubation period. These results suggest the progression of prion disease is influenced by the balance between replication and clearance of PrP^Sc^ in neurons.

## Introduction

Prion diseases are a group of transmissible, fatal neurodegenerative diseases, which include Creutzfeldt-Jakob disease in humans, bovine spongiform encephalopathy in cattle, and scrapie in sheep. The prion agent is comprised mainly, if not entirely, of PrP^Sc^ which is an abnormal isoform of the host encoded prion protein, PrP^C^ [1], [2], [3], [4], [5], [6]. Prion propagation is thought to occur in a two-step process where PrP^Sc^ first binds to PrP^C^ followed by a conformational conversion of PrP^C^ to PrP^Sc^ [7], [8], [9]. This conversion results in a change in physical properties of PrP^C^ that include an increase in β-pleated sheet content, decreased solubility in non-denaturing detergents and increased resistance to proteolytic degradation [3], [10], [11].

Prion strains are operationally defined by characteristic incubation periods and neuropathological features that are maintained upon experimental passage [12], [13]. The distribution of PrP^Sc^ in organs and neuronal populations can differ between strains, suggesting that PrP^Sc^ has a distinct strain-specific cellular tropism [14], [15], [16], [17]. The initial uptake of PrP^Sc^ by different cell-lines appears to be independent of the particular strain [18], [19] and suggests that cellular factors are responsible for prion strain tropism [17], [20], however, this has not been confirmed *in vivo* [21].

Prion strain diversity may be encoded by unique strain-specific conformations of PrP^Sc^ [15], [22], [23], [24], [25], [26]. Consistent with this, strain specific differences in the molecular weight of PrP^Sc^ following limited PK digestion, the relative resistance of PrP^Sc^ to degradation by PK, the relative alpha helical and beta sheet content of PrP^Sc^, the resistance of PrP^Sc^ to PK digestion in increasing concentrations of a protein denaturant (i.e. conformational stability), and the aggregation state of PrP^Sc^ have been observed [15], [22], [27], [28]. The mechanisms underlying how strain-specific conformations of PrP^Sc^ result in the distinct biological properties of disease are poorly understood.

The published reports on the relationship between the conformational stability of PrP^Sc^ and the length of the incubation period of disease between prion strains are contradictory. In murine prion strains and during adaptation of synthetic prions, a decrease in the conformational stability of PrP^Sc^ correlates with a corresponding decrease in the incubation period [5], [24], [29], [30]. One explanation for this observation is that a decrease of PrP^Sc^ stability increases PrP^Sc^ fragmentation resulting in an increase in agent replication that produces a correspondingly shorter incubation period [31], [32], [33]. Consistent with this, a decrease in Sup35 fiber stability corresponds to an increased rate of fibril fragmentation in yeast prions [33], [34]. These data contrast with what has been observed in hamster-adapted prion strains. Short incubation period prion strains have PrP^Sc^ that is conformationally more stable compared to PrP^Sc^ from strains with a relatively longer incubation periods in hamsters [27]. However, a direct comparison between PrP^Sc^ replication rate and conformational stability has not been investigated.

Both the prion strain and the cell type infected can influence the processing of PrP^Sc^. Studies of sheep infected with different prion strains, either naturally or experimentally, have identified strain-specific patterns of PrP^Sc^ truncation in both neurons and glia [35], [36]. Within a given strain the PrP^Sc^ truncation pattern can differ between glia and neurons suggesting that factors in addition to the conformation of PrP^Sc^ contribute to PrP^Sc^ truncation. While it is thought that replication in neurons is more important to disease development compared to glia, the effect of strain-specific processing of PrP^Sc^ in these cell types is less clear [37], [38], [39].

To better understand the strain specific relationship between the agent and the host, we evaluated PrP^Sc^ amplification efficiency, conformational stability of PrP^Sc^, and susceptibility of PrP^Sc^ to endogenous proteolytic processing *in vivo* in several cell types, of eight hamster-adapted prion strains. Our data indicate that short incubation period strains have correspondingly more efficient replication, a higher conformational stability, and intrasomal accumulation of PrP^Sc^ in neurons compared to long incubation period strains. These data suggest that the relationship between agent replication and clearance influence the progression of disease.

## Results

### The molecular weight and abundance of PrP^Sc^ from multiple hamster-adapted prion strains is similar

**Figure 1:**
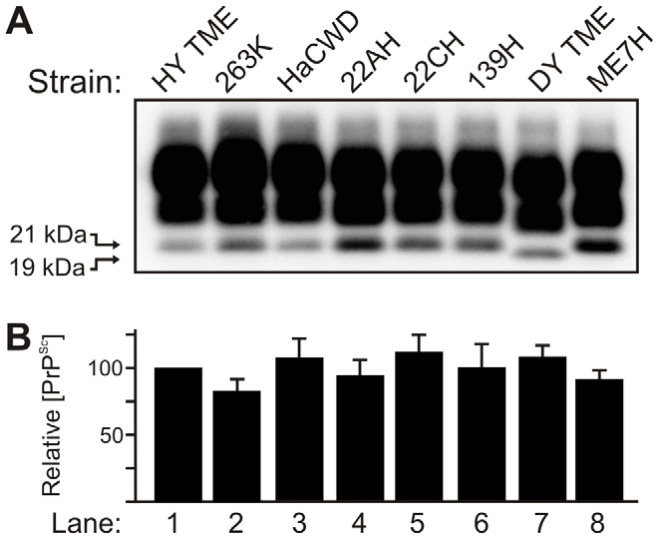
The molecular weight and abundance of brain-derived PrP^Sc^ of hamster-adapted hamster strains are similar. A) Western blot analysis and B) quantification (n = 4) of PrP^Sc^ from proteinase K digested brain homogenate of hamsters at terminal disease infected with either the HY TME, 263K, HaCWD, 22AH, 22CH, 139H, DY TME or ME7H agents. The migration of the 19 and 21 kDa unglycosylated PrP^Sc^ polypeptides is indicated on the left of panel A.

Brain tissue from hamsters at terminal disease infected with either the HY TME, 263K, HaCWD, 22AH, 22CH, 139H, DY TME or ME7H agents was digested with proteinase K and 250 µg equivalents were analyzed by Western blot (Figure 1). Western blot analysis of PrP^Sc^ indicated that the unglycosylated PrP^Sc^ glycoform of each strain migrated at 21 kDa with the exception of DY PrP^Sc^, which migrated at 19 kDa (Figure 1A, Table 1). The abundance of PrP^Sc^ was determined for each strain (n = 4) and there was less than a 25% difference in the abundance of PrP^Sc^ per µg brain equivalent for each prion strain analyzed (Figure 1B).

**Table 1:** Properties of hamster-adapted prion strains.

	Incubation period (days)		PrP^Sc^ properties					
Strain	i.c.	i.sc.	Migration	[Gdn-HCl]_1/2_	[SDS]_1/2_	Amplification coefficient	Source	Ref.
HY TME	65±3^a^	70±3	21 kDa	1.16±0.09	1.14±0.03	20	TME	[58]
263K	61±3	72±3	21 kDa	1.57±0.02	1.04±0.06	20	Scrapie	[62]
HaCWD	61±3	73±3	21 kDa	1.27±0.09	0.78±0.02	2	CWD	[63]
22AH	136±5	n.d.	21 kDa	1.02±0.02	0.53±0.04	0.02	Scrapie	[64]
22CH	161±3	n.d.	21 kDa	0.67±0.02	0.46±0.02	0.02	Scrapie	[64]
139H	159±3	198±3	21 kDa	0.76±0.05	0.50±0.01	0.02	Scrapie	[64]
DY TME	170±4	235±3	19 kDa	0.43±0.03	0.53±0.05	0.02	TME	[58]
ME7H	263±3	n.d.	21 kDa	0.59±0.03	0.44±0.02	0.02	Scrapie	[64]

a Mean ± SEM, n = 5.

n.d. – not done.

### Short incubation period strains replicate PrP^Sc^ more efficiently compared to PrP^Sc^ from long incubation period strains

**Figure 2:**
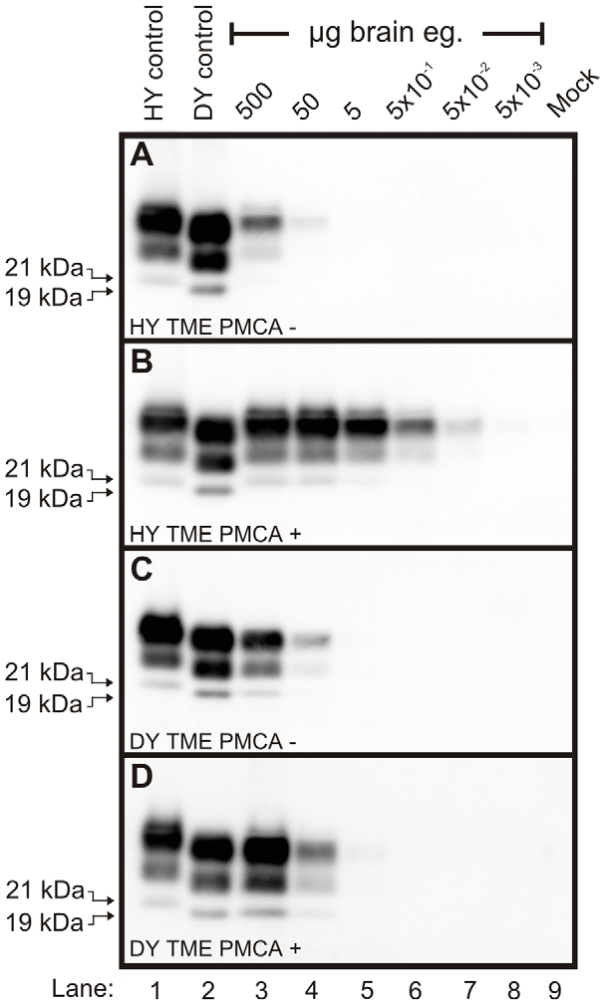
Kinetics of HY and DY PrP^Sc^ amplification correspond to differences in incubation period. Western blot analysis was performed on 10-fold serial dilutions of either HY TME (Panels A and B) or DY TME (Panels C and D) agent infected brain homogenate prior to (Panels A and C) or after one round of PMCA (Panels B and D). The migration of the 19 and 21 kDa unglycosylated PrP^Sc^ polypeptides is indicated on the left of each panel. Mock – mock infected negative control reaction.

To determine if differences exist in the rate of PrP^Sc^ replication between strains, protein misfolding cyclic amplification (PMCA) was performed on eight hamster-adapted prion strains. Brain homogenates were prepared from animals at the clinical stage of disease or from an uninfected (mock) negative control and serial 10-fold serial dilutions of these homogenates were analyzed by Western blot prior to (Figure 2A and 2C) or after one round of PMCA (Figure 2B and 2D). PMCA reactions that were initially seeded with 500 to 5×10^−2^ µg eq of HY TME infected brain homogenate resulted in detectable amplification of PrP^Sc^, but amplification was not detected in PMCA reactions seeded with lower concentrations of HY brain homogenate (Figure 2B). One round of PMCA using brain homogenate from DY TME infected animals amplified PrP^Sc^ to detectable levels in reactions that were initially seeded with 500 or 50 µg eq, but was not detected in PMCA reactions seeded with lower concentrations (Figure 2D). This was the general trend, as the short incubation period strains HY TME, 263K, and HaCWD resulted in detection of amplified PrP^Sc^ in reactions seeded with lower ug eq of brain homogenate compared to the longer incubation period strains 22AH, 22CH, 139H, DY TME, and ME7H (Figure S1, Table 1). These data demonstrate that the efficiency of PrP^Sc^ amplification corresponds with the incubation period for the prion strains that were analyzed.

### PrP^Sc^ from short incubation period strains is conformationally more stable than PrP^Sc^ from long incubation period strains

**Figure 3:**
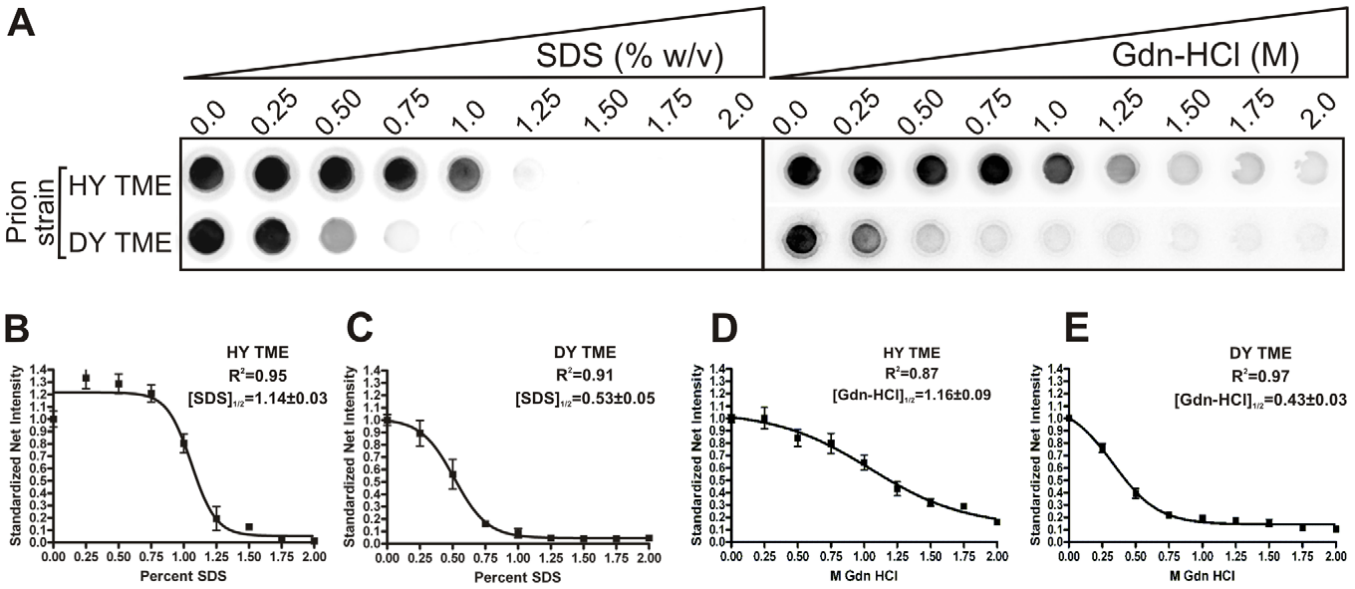
HY PrP^Sc^ is more conformationally stable than DY PrP^Sc^ in either Gdn-HCL or SDS. A) HY or DY TME infected brain homogenate was treated with increasing concentrations of either SDS or Gdn-HCL, digested with PK and the remaining PrP^Sc^ was detected using a 96 well immunoassay. The concentration of either SDS or Gdn-HCL required for a 50% reduction in PrP^Sc^ is greater for HY TME (panels B and D) compared to DY PrP^Sc^ (Panels C and E).

The conformational stability of PrP^Sc^ for each of the eight hamster-adapted prion strains was determined using either SDS or Gdn-HCl to denature PrP^Sc^. The [SDS]_1/2_ half values (% w/v) segregated into two groups corresponding to the incubation period of the strain. The short incubation period strains HY TME, 263K, and HaCWD have a [SDS]_1/2_ values of 1.14±0.03, 1.04±0.06 and 0.78±0.02 respectively compared to the long incubation period strains 22AH, 22CH, 139H, DY TME, and ME7H, which have [SDS]_1/2_ values of 0.53±0.04, 0.46±0.02, 0.50±0.01, 0.53±0.05 and 0.44±0.02 respectively (Table 1, Figure 3, Figure S2). Similarly, there was a corresponding decrease in the [Gdn-HCl]_1/2_ values with an increase in the incubation period. To demonstrate that the reduction in PrP^Sc^ was not due to an inhibition of PrP^Sc^ binding to the PVDF membrane due to the presence of SDS or Gdn-HCl, the PK digestion step of the conformational stability assay was omitted which resulted in the detection of PrP^Sc^ (data not shown). Overall, this data demonstrates that PrP^Sc^ from the short incubation period strains is more stable than PrP^Sc^ from the long incubation period strains (Table 1, Figure 3, Figure S2).

### Absence of PrP^Sc^ from the soma of neurons corresponds with increased survival times

**Figure 4:**
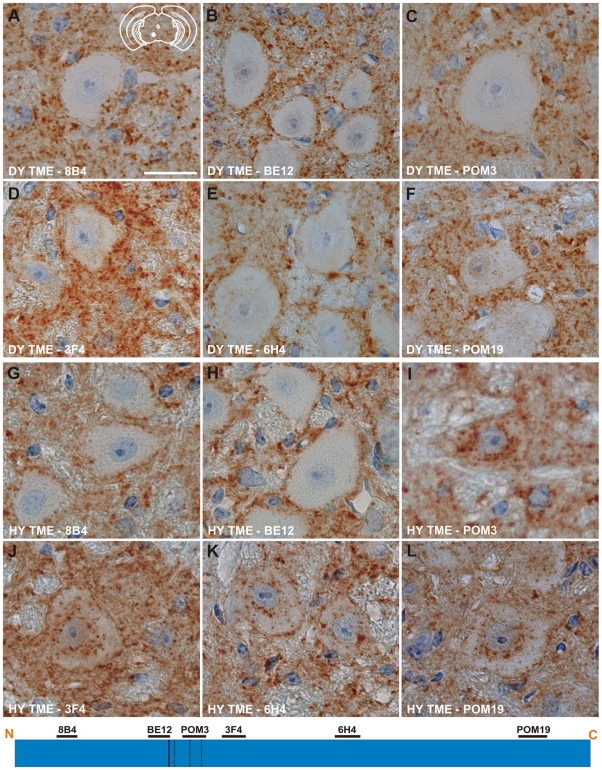
Strain specific differences in the clearance of PrP^Sc^ in neurons of hamsters infected with either the DY or HY TME agents. PrP^Sc^ immunohistochemistry was performed on CNS from hamsters infected with either the DY (panels A–F) or the HY TME (panels G–L) agents. PrP^Sc^ deposits are detected in the neuropil of hamsters infected with the long incubation period strain DY TME, however, PrP^Sc^ was rarely detected in the soma of neurons (panels A–F). In hamsters infected with the short incubation period strain HY TME, PrP^Sc^ is detected in the neuropil with all antibodies used (panels G–L). In contrast to the DY TME infected brain, HY PrP^Sc^ was detected in the somata of neurons with anti-PrP antibodies whose epitopes are C-terminal to the *in vitro* PK cleavage site (panels I–L). The yellow region in the schematic insets in panel A depict the location in the brain area that was imaged for every panel. The schematic at the bottom of the figure represents the location of the anti-PrP antibodies and the HY and DY PrP^Sc^ PK cleavage sites are depicted as solid and dashed lines, respectively. Scale bar, 50 µm.

Immunohistochemistry was performed on CNS tissue of hamsters using a panel of six monoclonal anti-PrP antibodies whose epitopes span the length of the hamster PrP protein (Table 2). Immunohistochemistry using this panel of six anti-PrP antibodies on mock-infected tissue sections containing red nucleus neurons failed to detect PrP^Sc^, indicating the specificity of the antibodies for PrP^Sc^ (Figure S3). PrP^Sc^ deposits were detected perineuronally and within the neuropil of the red nucleus with every anti-PrP antibody tested in animals infected in the sciatic nerve with the DY TME agent at clinical disease suggesting the presence of full length PrP^Sc^ (Figure 4A to 4F). Within the soma of neurons, the three antibodies whose epitopes are at the N terminus of PrP (8B4, BE12, and POM3) failed to detect PrP^Sc^ deposition (Figure 4A to 4C). The three antibodies whose epitopes are located toward the C-terminal region of PrP (3F4, 6H4, and POM19) occasionally identified PrP^Sc^ deposition within the soma of these neurons, however, these DY PrP^Sc^ deposits appeared diffuse and faint compared to the PrP^Sc^ immunoreactivity in the neuropil (Figure 4D to 4F, Table S1). This same pattern of PrP^Sc^ distribution was also observed in VMNs of the lumbar spinal cord, and the neurons in the interposed nucleus, red nucleus and hind limb motor cortex throughout the course of disease and in animals inoculated by the i.c. route at clinical disease (data not shown). The distribution of PrP^Sc^ in the red nucleus of hamsters i.c. inoculated with the long incubation period strains 22AH, 22CH, 139H or ME7H was indistinguishable from DY TME agent infected animals (Figure S4, Table S1).

**Table 2:** Anti-PrP antibodies used for immunohistochemistry.

Antibody	Dilution	Species^a^	Region^b^	Sequence^c^	Ref.
8B4	1∶400	mouse	N-terminal	_35_ GSRYPGQGSPG_45_	[65]
BE12	1∶400	sheep	N-terminal	_23_ LCK……GGG_90_	[66]
POM3	1∶100	mouse	C-terminal	_95_ HNQWNK_100_	[67]
3F4	1∶600	hamster	C-terminal	_109_ MKHM_112_	[68]
6H4	1∶400	human	C-terminal	_144_ DYEDRYYRE_152_	[69]
POM19	1∶400	mouse	C-terminal	_218_ YQKE_221_	[67]

a Species that anti-PrP antibody is generated against.

b In relation to the proteinase K digestion site of HY PrP^Sc^.

c Prion protein sequence epitope.

To extend these studies on a short incubation period strains, we performed PrP^Sc^ immunohistochemistry on the red nucleus from animals infected in the sciatic nerve with the HY TME agent at clinical disease to ensure a direct comparison could be made with DY TME agent infected animals. The deposition of PrP^Sc^ in the neuropil and soma of neurons was similar to what was observed following infection with the DY TME agent and the other the long incubation period strains using the anti-PrP antibodies whose epitopes are located N-terminal to the HY PrP^Sc^ PK cleavage site (Figure 4, Panels G–H). However, when using antibodies located C-terminal to the HY PrP^Sc^ PK cleavage site, intrasomal and perinuclear PrP^Sc^ deposition was detected that was similar in intensity to PrP^Sc^ deposition in the neuropil (Figure 4I to 4L, Table S1). This HY TME specific pattern of PrP^Sc^ deposition was observed in animals inoculated by either the sciatic nerve or i.c. routes of inoculation at early and late time points post-infection and in the same brain regions that were examined in the DY TME infected animals. This pattern of HY PrP^Sc^ truncation was also observed in animals inoculated with the short incubation period strains 263K and HaCWD by the i.c. route at clinical disease (Figure S4, Table S1). These data reveal similarities in the PrP^Sc^ deposition patterns in the neuropil and somata of neurons of animals infected with either long or short incubation period strains.

### Truncation of HY PrP^Sc^ within the soma of neurons

**Figure 5:**
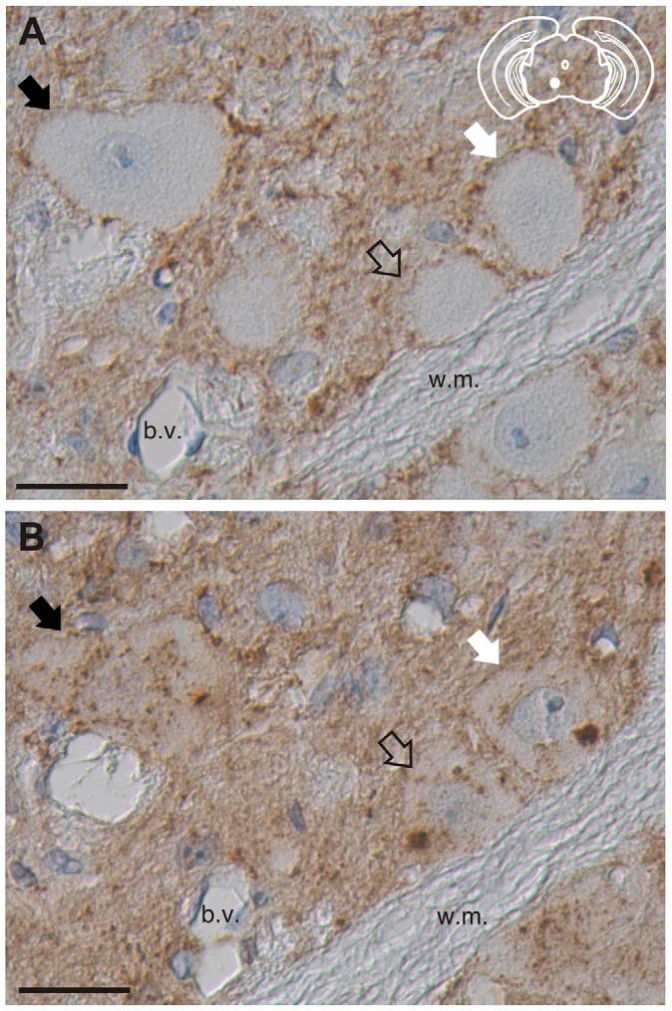
Truncation of the N-terminus of HY PrP^Sc^ within neurons. PrP^Sc^ immunohistochemistry was performed on serial sections with either an anti-PrP antibody whose epitope is either A) N-terminal (BE12) or B) C-terminal (POM3) to the HY PrP^Sc^ PK cleavage site. Arrows indicate the same neurons in panels A and B. The yellow region in the schematic insets depict the location in the brain area that was imaged in each panel. Abbreviations: b.v., blood vessels; w.m., white matter. Scale bar, 50 µm.

The absence of PrP^Sc^ immunoreactivity using antibodies directed against the N-terminal regions of PrP^Sc^ suggests that truncated PrP^Sc^ is present in these cells. To investigate this possibility, serial sections of red nucleus from clinically-ill HY TME infected hamsters were processed using either the BE12 or POM3 antibodies whose epitopes are N-terminal and C-terminal to the HY PrP^Sc^ PK cleavage site respectively. Fiduciary marks, such as blood vessels and white matter tracts, were used to increase the likelihood that the same neurons were analyzed in both sections. The BE12 antibody detected punctate HY PrP^Sc^ deposits in the neuropil and perineuronally in the red nucleus but failed to detect intrasomal PrP^Sc^ (Figure 5A). However, the POM3 antibody detected coarse, intrasomal PrP^Sc^ deposits in these same three neurons (Figure 5B, arrowheads). Additionally, the intrasomal PrP^Sc^ formed large perinuclear aggregates (Figure 5B). This demonstrates that the loss of N-terminal epitopes of PrP^Sc^ and the aggregation of the C-terminal PrP^Sc^ fragments occurs within the same neuron.

### Processing of PrP^Sc^ in astrocytes and microglia is nearly uniform between strains

The deposition of PrP^Sc^ in astrocytes and microglia was investigated using the same panel of anti-PrP antibodies in combination with anti-GFAP and anti-Iba-1, which label astrocytes and microglia, respectively. As negative controls, reactive astrogliosis, microgliosis or PrP^Sc^ immunoreactivity was not detected in mock-infected animals (Figure S5A to S5F). Additionally, non-specific binding of the monoclonal antibodies or the fluorescently conjugated secondary antibodies was not detected (Figure S5G and S5H).

**Figure 6:**
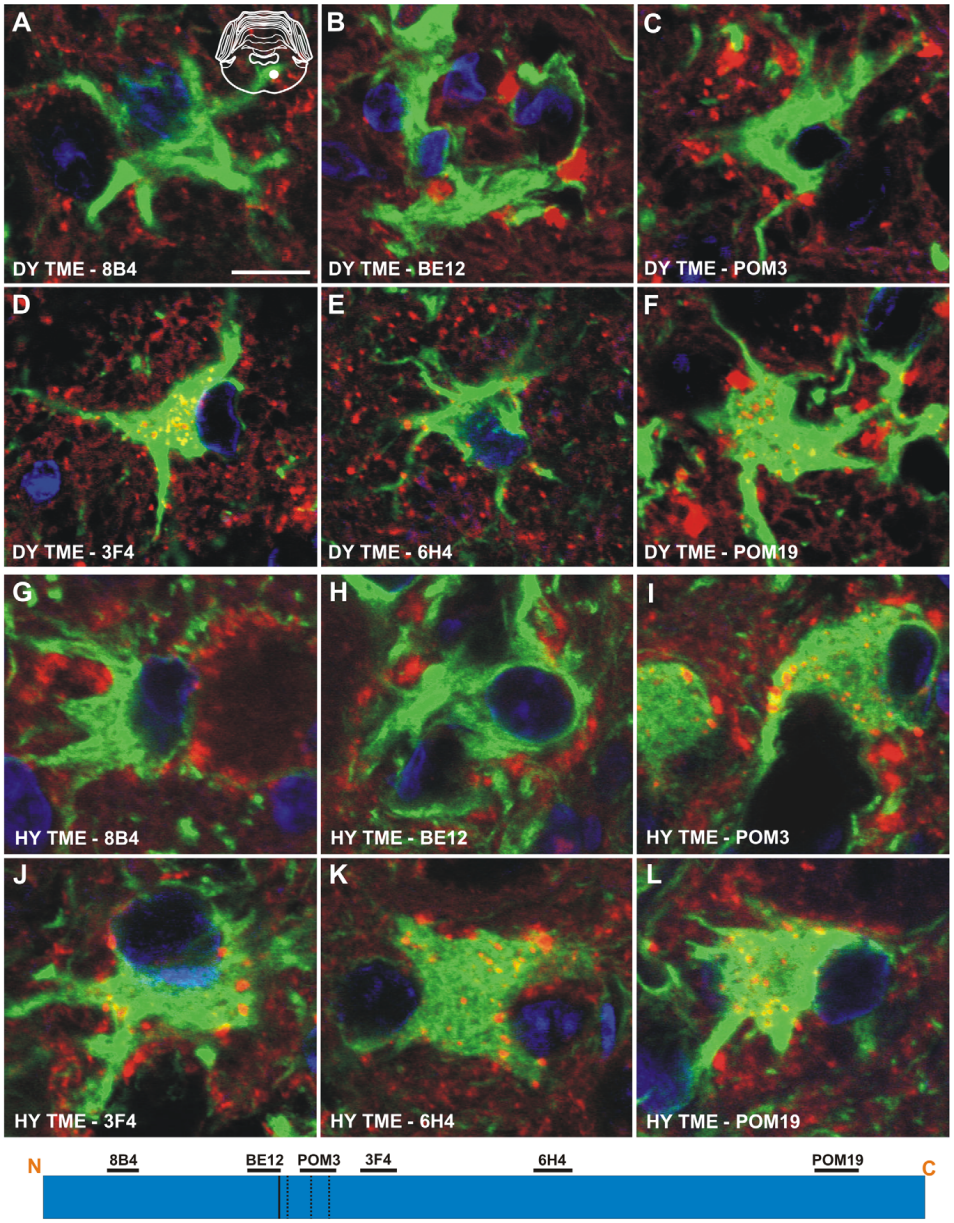
Strain-specific truncation of PrP^Sc^ in astrocytes of hamsters infected with either the DY or HY TME agents. Dual immunofluorescence was performed on brains of DY TME (panels A–F) or HY TME (panels G–L) infected animals using antibodies directed against PrP (red fluorescence) and GFAP (green fluorescence). Dual PrP/GFAP immunofluorescence was performed on the reticular formation from DY TME (A–F) or HY TME (G–L) agent infected hamsters at the clinical stage of disease. The solid white circle located in the schematic inset is the location of the photographed images within the reticular formation. The schematic at the bottom of the figure represents the location of the anti-PrP antibodies and the HY and DY PrP^Sc^ PK cleavage sites are depicted as solid and dashed lines, respectively. The HY and DY PrP^Sc^ PK cleavage sites are also depicted as the solid and dashed lines respectively. Scale bar, 10 µm.

**Figure 7:**
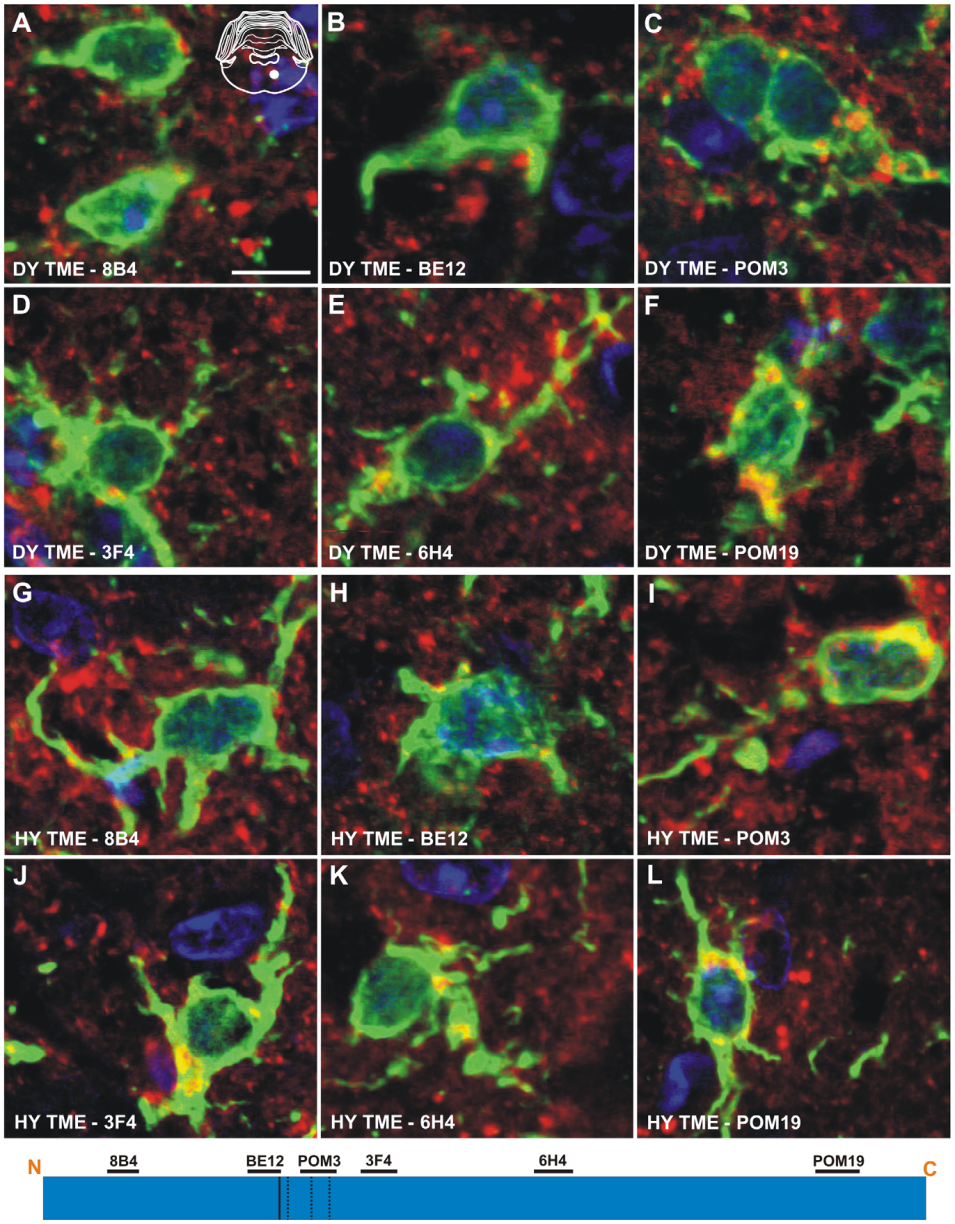
Identical processing of PrP^Sc^ in microglia of hamsters infected with either the DY or HY TME agents. Double immunofluorescence was performed using antibodies directed against PrP (red fluorescence) and Iba-1 (green fluorescence). (A–F) Immunofluorescence in the reticular formation of DY TME infected hamsters at the clinical stage of disease. (G–L) Immunofluorescence in the reticular formation of HY TME infected hamsters at the clinical stage of disease. The solid white circle located in the schematic inset is the location of the brain area that was imaged in each panel. The schematic at the bottom of the figure represents the location of the anti-PrP antibodies and the HY and DY PrP^Sc^ PK cleavage sites are depicted as solid and dashed lines, respectively. Scale bar, 10 µm.

The anti-PrP antibodies 8B4, BE12, and POM3 failed to detect PrP^Sc^ within astrocytes (Figure 6A to 6C), while the antibodies 3F4, 6H4, and POM19 detected coarse punctate PrP^Sc^ deposits in astrocytes of hamsters infected with the DY TME agent at clinical disease (Figure 6D to 6F, Table S1). The anti-PrP antibodies POM3, 3F4, 6H4, and POM19 detected PrP^Sc^ within astrocytes, while the antibodies 8B4 and BE12 failed to detect PrP^Sc^ within astrocytes of hamsters infected with the HY TME agent at clinical disease (Figure 6G to 6L, Table S1). The same PrP^Sc^ truncation pattern detected in astrocytes of HY TME infected animals was also observed in animals infected with the 263K, HaCWD, 22AH, 22CH, 139H or ME7 agents (Figure S6, Table S1). The anti-PrP antibodies 8B4 and BE12 failed to detect PrP^Sc^ in microglia, while the anti-PrP antibodies POM3, 3F4, 6H4, and POM19 detected coarse punctate PrP^Sc^ deposits within these cells (Figure 7, Figure S7, Table S1).

## Discussion

Here we show that short incubation period strains have a more stable PrP^Sc^ conformation when compared to long incubation period strains. PrP^Sc^ conformational stability assays using either Gdn-HCl or SDS as the denaturant found the same relationship between the conformational stability of PrP^Sc^ and incubation period of disease indicating that this relationship is independent of the denaturant used (Table 1). This relationship between PrP^Sc^ conformational stability and incubation period is consistent with previous work examining the conformational stability of purified PrP^Sc^ from hamster-adapted prion strains [27]. In contrast to what is observed in hamsters, a decrease in the PrP^Sc^ conformational stability correlates with a reduction in the incubation period in mice [24], [29], [40]. The results in murine systems suggest that decreasing PrP^Sc^ stability increases the fragmentation of PrP^Sc^ therefore allowing in the generation of more PrP^Sc^ surfaces for PrP^C^ to bind resulting in an increased rate of PrP^Sc^ formation and subsequently shortening of the incubation period. Consistent with this hypothesis, studies examining Sup35, PrP, Tau, α-synuclein, and ß-amyloid demonstrate that less stable fibrils have a higher propensity to undergo breakage, thereby creating new seeds for conversion [33], [34], [41], [42], [43], [44], [45].

The PrP^Sc^ conformational stability data presented here suggest that conformationally stable PrP^Sc^ may also be more susceptible to fragmentation. SDS, like Gdn-HCl, can increase the susceptibility of PrP^Sc^ to PK digestion and inactivate the agent [27], [46], [47]. Since treatment of PrP^Sc^ that is enriched using detergent extraction and ultracentrifugation with SDS results in the disaggregation of PrP^Sc^ and the production of smaller PrP^Sc^ particles, SDS can affect the aggregation state of PrP^Sc^ [32], [48]. Therefore, the higher concentration of SDS required to increase the susceptibility of PrP^Sc^ to PK digestion of short incubation period strains may be due to increased PrP^Sc^ particle size compared to long incubation period strains.

Short incubation period strains have more efficient PrP^Sc^ amplification compared to long incubation period strains. We used PMCA to determine the relative efficiency of PrP^Sc^ conversion between hamster strains. We have previously shown that PMCA of HY and DY TME recapitulates the strain-specific properties of PrP^Sc^ and faithfully replicates the HY and DY TME agents [49]. In examining the eight hamster strains we found that the efficiency of PrP^Sc^ amplification correlated with the strains respective incubation periods, as the strains with more efficiently replicating PrP^Sc^ had a shorter incubation period compared to long incubation period strains (Table 1). This is consistent with cell-free conversion experiments that demonstrated a faster rate of HY PrP^Sc^ synthesis compared to the rate of DY PrP^Sc^ synthesis [50]. The data presented here also indicate that conformationally more stable PrP^Sc^ amplifies more efficiently compared to less stable PrP^Sc^. Interestingly, the short incubation period strain HaCWD has conformationally less stable PrP^Sc^ in SDS compared to 263K and HY PrP^Sc^ which corresponded with a lower amplification efficiency compared to the two other short incubation period strains. A possible explanation for the increased amplification efficiency of PrP^Sc^ from the short incubation period strains is that this PrP^Sc^ is more likely to fragment due to its large PrP^Sc^ particle size compared to the longer incubation period strains used in this study. Alternatively, a minor subpopulation of PrP^Sc^ that is conformationally less stable may be responsible for the highly efficient PrP^Sc^ replication that was observed. This conformationally less stable subpopulation may be masked by an excess of conformationally more stable PrP^Sc^ that replicates with lower efficiency [32], [51], [52].

**Figure 8:**
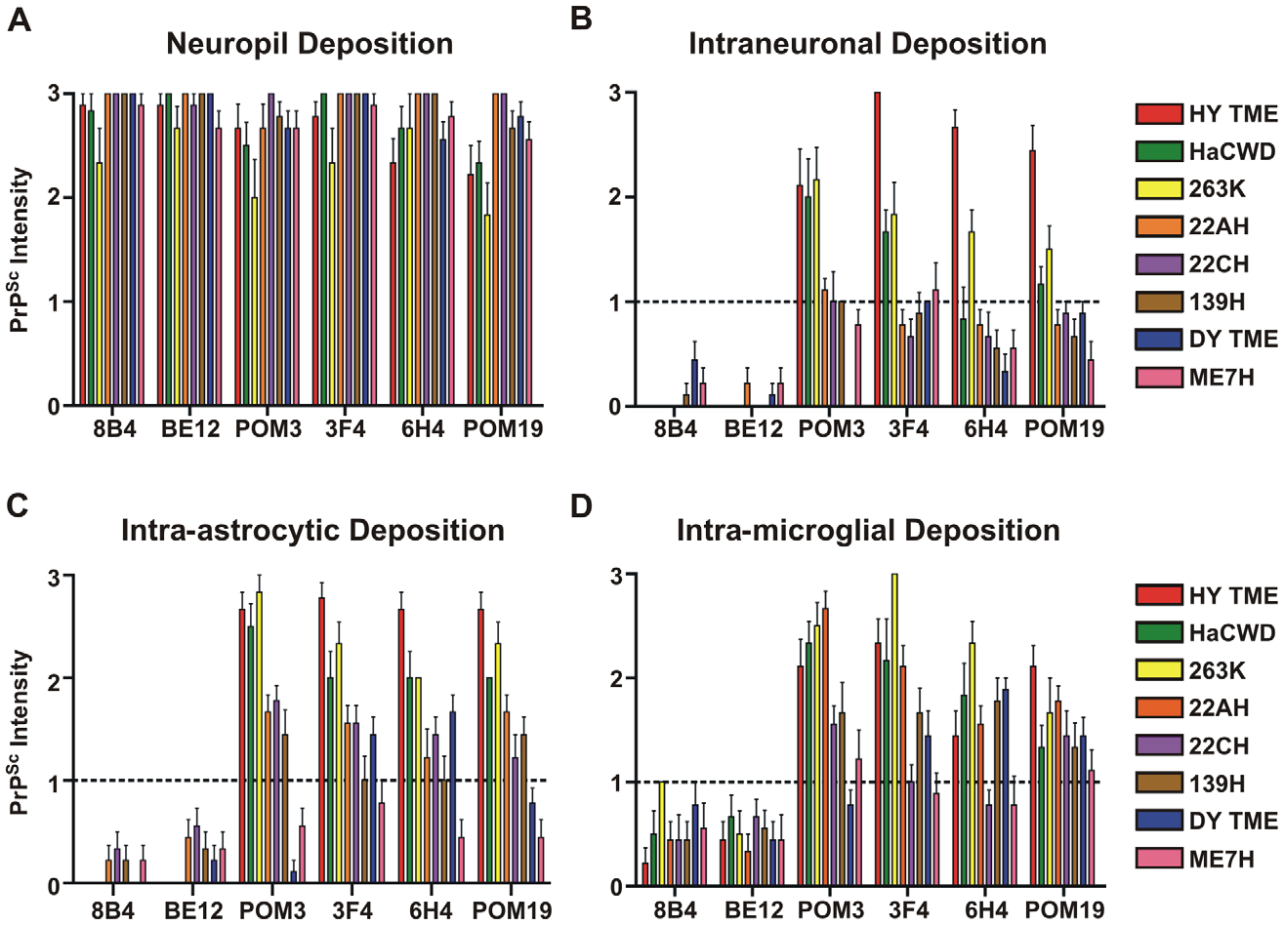
Strain-specific processing of PrP^Sc^ in neurons and glia. The intensity of PrP^Sc^ immunoreactivity for each hamster-adapted prion strain was scored following immunohistochemistry using either 8B4, BE12, POM3, 3F4, 6H4 or POM19 anti-PrP antibodies. PrP^Sc^ deposition was scored in the neuropil (A), within neurons (B), astrocytes (C), and microglia (D). Bars represent mean PrP^Sc^ intensity values and lines represent standard error.

**Figure 9:**
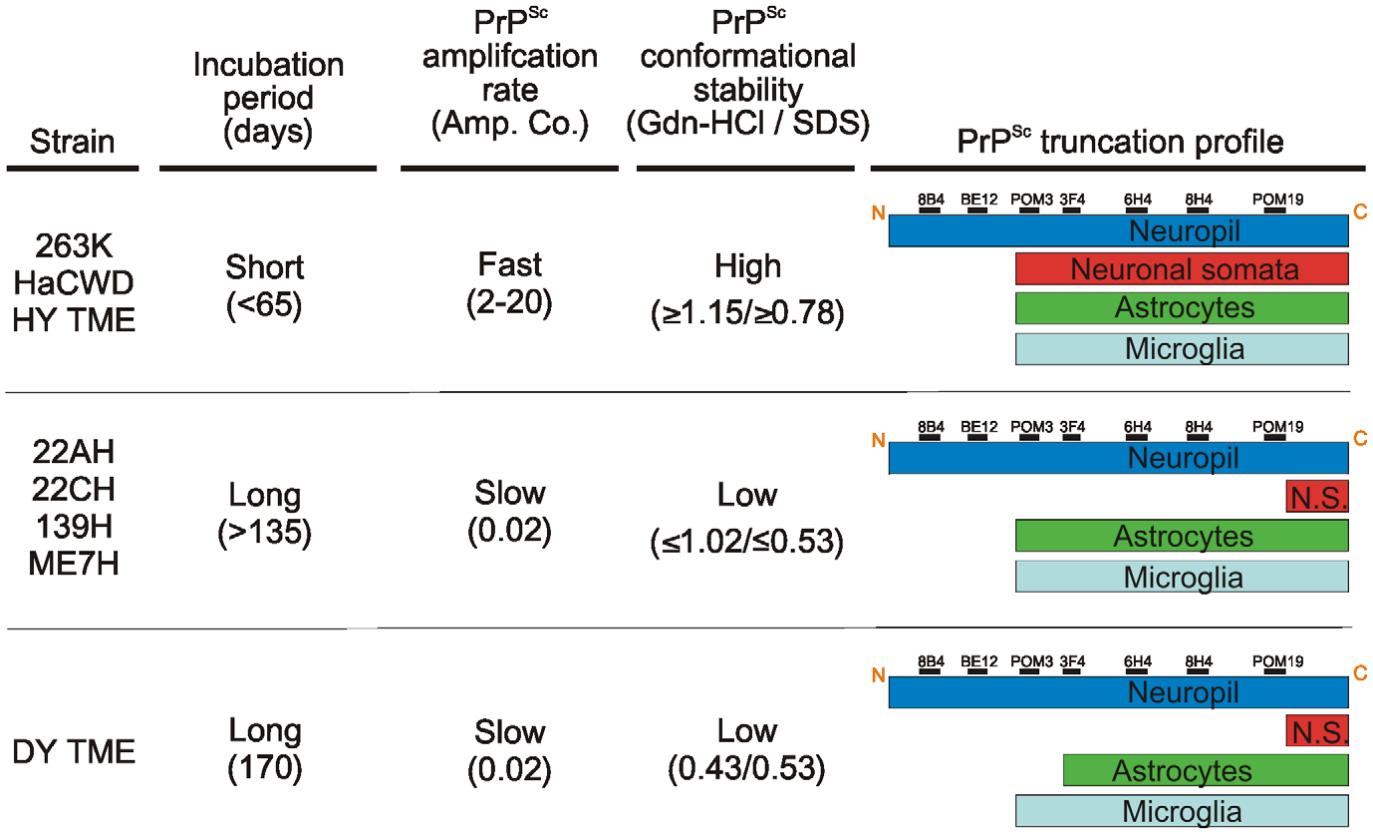
The relationship between PrP^Sc^ replication, stability and deposition in neurons is predictive of the incubation period of disease. The prion strains were grouped according to commonalities in incubation period, PrP^Sc^ amplification rate, PrP^Sc^ conformational stability and PrP^Sc^ truncation profile in neurons and glia. N.S. – neuronal somata.

Strain and cell-specific variations in the proteolytic processing of PrP^Sc^ have been observed in both brain tissue and cultured cells [36], [53], [54], [55], [56]. The results presented here are consistent with these findings and additionally suggest a relationship between the extent of truncation of PrP^Sc^ within the soma of neurons and the strains respective incubation periods. The short incubation period strains, HY TME, 263K, and HaCWD, contained a longer portion of C-terminal protein intact and a large punctate deposition of PrP^Sc^ within the soma of neurons, compared to the longer incubation period strains suggesting a strain-specific clearance of PrP^Sc^ (Figures 8, Figure 9). Furthermore, the low immunoreactivity of PrP^Sc^ in ME7H infected animals observed with all six anti-PrP antibodies and within all three cell types examined (Figure 8, Table S1) may represent the more efficient clearance of PrP^Sc^ in both neurons and glia for this particular strain and account for its significantly longer incubation period. However, we cannot exclude the possibility that the inability to detect intense PrP^Sc^ immunoreactivity in the soma of neurons from animals inoculated with the long incubation period strains is due to a failure of PrP^Sc^ transport to the soma. This strain-specific truncation pattern was only observed in neurons, as the same N-terminally truncated PrP^Sc^ species was detected in astrocytes and microglia for all strains examined, with the lone exception of the loss of the POM3 epitope from DY PrP^Sc^ within astrocytes (Figure 8, Figure 9). These data support the hypothesis that direct infection of neurons leads to more rapid death of neurons resulting in shorter incubation periods, compared to indirect neuronal death via infection of astrocytes and microglia [37], [38], [57].

The results presented here suggest the strain-encoded relationship between PrP^Sc^ replication, stability and processing in neurons is predictive of the incubation period of disease (Figure 9). Here we show that strains with a short incubation period have conformationally stable PrP^Sc^ that replicates efficiently. The fast replication and stable PrP^Sc^ may be responsible for the accumulation of PrP^Sc^ in the soma of neurons resulting in a shorter incubation period. The long incubation period strains displayed relatively less efficient PrP^Sc^ replication and less stable PrP^Sc^. In these strains, the combination of a slower replicating agent and PrP^Sc^ that is less stable may result in neurons to be able to more effectively cleared of PrP^Sc^ resulting in longer incubation periods.

## Materials and Methods

### Ethics statement

All procedures involving animals were approved by the Creighton University Institutional Animal Care and Use Committee and were in compliance with the *Guide for the Care and Use of Laboratory Animals*.

### Animal inoculations

Sciatic nerve and intracerebral inoculations of the HY or DY TME agents were performed on male Syrian golden hamsters (Harlan-Sprague-Dawley, Indianapolis, IN) as previously described [21]. Groups of five hamsters were inoculated in the sciatic nerve or intracerebrally with 1 or 25 µl, respectively, of a 1% (wt/vol) brain homogenate from animals at the terminal stage of disease infected with either the HY TME, DY TME, 263K, HaCWD, 22AH, 22CH, 139H, or ME7H agents. Hamsters were observed three times per week for the onset of clinical signs as described previously [58]. Incubation period was calculated as the number of days between inoculation and onset of clinical signs.

### Tissue collection

Tissue from infected and mock-infected hamsters was collected for either immunohistochemistry (IHC) or Western blot analysis. For IHC analysis animals were anesthetized with isoflurane and perfused transcardially with 50 ml of 0.01 M Dulbecco's phosphate-buffered saline followed by 75 ml of McLean's paraformaldehyde-lysine-periodate (PLP) fixative as previously described [21], [59]. Brain was immediately removed and placed in PLP for 5 to 7 h at room temperature prior to paraffin processing. For Western blot analysis, animals were sacrificed by CO_2_ asphyxiation, and the brain was rapidly removed and flash frozen and stored at −80°C.

### Western blot analysis

Brain tissue and spinal cord tissue were homogenized to 10% w/v in Dulbecco's Phosphate Buffered Saline (DPBS) without Ca++ or Mg++ (Mediatech, Herndon, VA) containing protease inhibitors (Roche Diagnostics Corporation, Indianapolis, IN) by passing the tissue through a 26 g needle, followed by a 30 second incubation in a cup horn sonicator (Fisher Scientific, Atlanta, GA). The tissue was diluted to 5% w/v in DPBS containing proteinase K (PK) at a final concentration of 1 U/ml (Roche Diagnostics Corporation, Indianapolis, IN) and incubated at 37°C for 1 hour with constant agitation. The PK digestion was terminated by incubating the samples at 100°C for 10 minutes. SDS-PAGE and Western blot analysis were performed as described previously [49] using the anti-PrP antibody 3F4 (1∶600; Chemicon; Billerica, MA). The blot was developed with Pierce Supersignal West Femto Maximum Sensitivity Substrate according to manufactures instructions (Pierce, Rockford, IL) and imaged in the linear range of detection on a Kodak 4000R Imaging Station (Kodak, Rochester, NY) and analysis was performed using Kodak Molecular Imaging Software v.5.0.1.27 (New Haven, CT) as described previously [49].

### Protein misfolding cyclic amplification

Protein misfolding cyclic amplification (PMCA) was performed as previously described [1], [49]. Briefly, uninfected brain was homogenized to 10% (w/v) in ice-cold conversion buffer [phosphate buffer saline (pH 7.4) containing 5 mM EDTA, 1% v/v Triton X-100, and complete protease inhibitor tablet (Roche Diagnostics, Mannheim, Germany)] using a Tenbroeck tissue grinder (Vineland, NJ). The brain homogenate was centrifuged at 500× g for 30 seconds and the supernatant was stored at −80°C. PMCA was performed with a Misonix 3000 sonicator (Farmingdale, NY) with the sonicator output set to level 6 with an average output of 156 watts for each sonication cycle. All PMCA reactions were replicated in triplicate. One round of PMCA consisted of 144 cycles of a five-second sonication followed by a ten-minute incubation at 37°C. Before each PMCA round, an aliquot was placed at −80°C as an unsonicated control. Samples seeded with prion-infected brain homogenate were replicated using a minimum of three individual hamster brains to control for variation between animals. Samples containing uninfected brain homogenate in conversion buffer alone were included in every round of PMCA as a negative control. The amplification efficiency was calculated as the reciprocal of the µg equivalent of last dilution of prion-infected brain homogenate that resulted in detectable amplified PrP^Sc^ following one round of PMCA.

### Conformation stability assay

Brain homogenates [7.5% (w/v)] were diluted in either SDS (Fischer Scientific, Atlanta GA) to a final concentration of 0, 0.25, 0.5, 0.75, 1, 1.25, 1.5, 1.75, or 2% (w/v) or in Gdn-HCl (Sigma-Aldrich, St. Louis, MO) to a final concentration of 0, 0.25, 0.5, 0.75, 1, 1.25, 1.5, 1.75, or 2 molar and were immediately heated at 70°C for 10 min. Proteinase K was added to 0.0625 U/ml (Roche Diagnostics, Indianapolis, IN) and the samples were incubated at 37°C for 15 min while shaking. All samples were brought to 200 µl in DPBS and the concentration of PrP^Sc^ was determined using a 96-well immunoassay as described previously [60]. Gdn-HCL and SDS treated samples were performed in quintuplicate. Serial two-fold dilutions of brain homogenate each strain were performed in triplicate to ensure that the PrP^Sc^ levels of the SDS or Gdn-HCl treated samples were in the linear range of PrP^Sc^ detection. Denaturation curves were generated by dividing the intensity of all samples by the average intensity of the 0% SDS or Gdn-HCl samples. A sigmoidal dose response curve with variable slope was fitted to the standardized values (Prism statistical software, GraphPad, La Jolla, CA). The [SDS]_1/2_ and [Gdn-HCl]_1/2_ values is the percentage of SDS or molarity of Gdn-HCl required for a 50% reduction in the PrP^Sc^ signal intensity.

### PrP^Sc^ immunohistochemistry

PrP^Sc^ IHC was performed as previously described [21], [59]. Briefly, 7 µm tissue sections were deparaffinized and incubated in 95% formic acid (Sigma-Aldrich, St.Louis, MO) followed by blocking of endogenous peroxidases by immersion in 0.3% H_2_O_2_ in methanol. Following blocking of non-specific staining with 10% horse serum, sections were incubated overnight with an anti-PrP antibody (Table 2) at 4°C. The sections were then incubated with a biotinylated horse anti-mouse immunoglobulin G conjugate and subsequent incubation with the ABC-horseradish peroxidase elite (Vector Laboratories, Burlingame, CA) staining kit. Sections were developed using 0.05% w/v 3,3′-diaminobenzidine (Sigma-Aldrich, St. Louis, MO) in tris-buffered saline containing 0.0015% H_2_O_2_ and counterstained with hematoxylin (Richard Allen Scientific, Kalamazoo, MI). Microscopy was performed using a Nikon i80 microscope (Nikon, Melville, NY) and images were captured using DigiFire camera and ImageSys digital imaging software (Soft Imaging Systems, GmbH) and processed using Adobe Photoshop CS2 v9.0.1 (Adobe Systems Inc., San Jose, CA).

For double immunofluorescence, tissue sections were deparaffinized and treated with 95% formic acid as described above. The tissue sections were blocked with 10% goat serum in tris-buffered saline for 30 minutes at room temperature, followed by overnight incubation at 4°C with the same panel of anti-PrP monoclonal antibodies (Table 2) and anti-glial fibrillary acidic protein (GFAP; 1∶16,000; Dako; Carpinteria, CA) or anti-ionized calcium binding adaptor molecule 1 (Iba-1; 1∶500; Abcam; Cambridge, MA). Sections were then incubated with both Alexa Fluor goat anti-mouse 546 and Alexa Fluor goat anti-rabbit 488 (1∶500; Invitrogen; Carlsbad, CA) secondary antibodies for one hour at room temperature. Slides were cover slipped using ProLong Gold antifade reagent with DAPI (Invitrogen; Carlsbad, CA).

### Confocal laser scanning microscopy

Fluorescent images were captured on a Zeiss LSM 510 META NLO confocal scanning system (Carl Zeiss Jena; Jena, Germany) using a Plan Neo 40× 1.3-NA DIC oil objective. Excitation of the Alexa Fluor antibodies and DAPI was achieved using an Argon laser at 488 nm, a Helium Neon laser at 543 nm, and a Coherent Chameleon near infrared tunable Ti:Sapphire laser. To increase the signal to noise ratio, each line was scanned 4 times and averaged. The pinhole aperture for each channel was adjusted so that an optical slice of 1.0 µm was imaged. In the profile view for each image, the line tool was used to draw an arbitrary line, and the relative fluorescent intensities along that line were compared to determine intracellular staining.

### Semi-quantitative measurement of PrP^Sc^ immunoreactivity

Semi-quantitative measurements of PrP^Sc^ immunoreactivity was performed as previously described [61]. Briefly, captured images were randomized and the relative magnitude of neuropil, intraneuronal, intra-astrocytic, and intra-migroglial PrP^Sc^ immunoreactivity was classified as absent (0), slight (1), moderate (2), or striking (3) from a minimum of 6 observations by three independent observers. The PrP^Sc^ immunoreactivity scores were compared between strains and cell types and analyzed by two-way analysis of variance and Bonferroni post-tests for statistical significance (p<0.05). These tests were performed using the Prism 4.0 (for Macintosh) software program (GraphPad Software, Inc., San Diego, CA).

## Supporting Information

Figure S1 PMCA replication efficiency of hamster adapted prion strains. Western blot analysis of PrP^Sc^ following one round of PMCA that was performed on 10 fold serial dilutions of brain homogenate from hamster infected with either the (A) 263K, (B) HaCWD, (C) 22AH, (D) 22CH, (E) 139H, or (F) ME7H agents. A mock infected negative control was included in every experiment. The migration of the 19 and 21 kDa unglycosylated PrP^Sc^ polypeptides is indicated on the left of each panel.
(5.76 MB TIF)

Figure S2 PrP^Sc^ conformational stability assays for multiple hamster-adapted prion strains. A) Brain homogenate from prion-infected hamsters were subject to incubation with increasing concentrations of either SDS or Gdn-HCl, digested with PK and the remaining PrP^Sc^ was detected using a 96-well immunoassay. The corresponding [SDS]_1/2_ and [Gdn-HCl]_1/2_ values were calculated from hamsters infected with either the (B,D) 263K, (C,E) HaCWD, (F,H) 22AH, (G,I) 22CH, (J,L) 139H, or (K,M) ME7H agents.
(1.15 MB TIF)

Figure S3 Specificity of anti-PrP antibodies for PrP^Sc^ immunodetection in the CNS of hamsters. PrP^Sc^ immunohistochemistry was performed on sections of red nucleus of a mock-inoculated animal using the anti-PrP antibodies (A) 8b4, (B) BE12, (C) POM 3, (D) 3F4, (E) 6H4, and (F) POM19 whose epitopes span from the N-terminal to C-terminal of PrP (Table 2). Scale bar, 50 µm.
(7.64 MB TIF)

Figure S4 Intrasomal deposition of PrP^Sc^ in neurons is a property of short incubation period strains in hamsters. PrP^Sc^ immunohistochemistry was performed on CNS tissue of hamsters at the clinical stage of disease following infection with either the 263K (A–F), HaCWD (G–L), 22AH (M–R), 22CH (S–X), 139H (Y–DD), or ME7H (EE–JJ) agents using the anti-PrP antibodies 8B4 (A, G, M, S, Y, EE), BE12 (B, H, N, T, Z, FF), POM 3(C, I, O, U, AA, GG), 3F4 (D, J, P, V, BB, HH), 6H4 (E, K, Q, W, CC, II) or POM 19 (F, L, R, X, DD, JJ). The schematic at the bottom of the figure represents the location of the anti-PrP antibodies and the HY and DY PrP^Sc^ PK cleavage sites are depicted as solid and dashed lines, respectively. Scale bar, 50 µm.
(9.06 MB TIF)

Figure S5 Specificity of immunolabeling and criteria of immunolabel co-localization. PrP^Sc^ immunofluorescence was performed on the reticular formation of a negative control mock-inoculated animal using the anti-PrP antibodies (A) 8b4, (B) BE12, (C) POM 3, (D) 3F4, (E) 6H4, or (F) POM19 and antibodies directed against (G) GFAP or (H) Iba-1. Non-specific binding of the monoclonal antibodies or fluorescently conjugated secondary antibodies was discounted by switching the appropriate secondary antibodies for (I, K) PrP, (J) GFAP, or (L) Iba-1. To determine co-localization of PrP^Sc^ within astrocytes or microglia using confocal microscopy, the relative fluorescence intensities of GFAP (M) and PrP^Sc^ (N) from the same 1 µm optical slice was merged (O) and a the relative intensities of the GFAP and PrP^Sc^ signals were determined along a line through the length of the cell (P). The solid white circle located in the schematic inset is the location of the photographed images within the reticular formation. Scale bar, 10 µm.
(6.42 MB TIF)

Figure S6 Similar N-terminal truncation of PrP^Sc^ in astrocytes of hamster-adapted strains. Dual fluorescence PrP^Sc^/GFAP immunohistochemistry was performed on CNS tissue of hamsters at the clinical stage of disease following infection with either the 263K (A–F), HaCWD (G–L), 22AH (M–R), 22CH (S–X), 139H (Y–DD), or ME7H (EE–JJ) agents using the anti-PrP antibodies 8B4 (A, G, M, S, Y, EE), BE12 (B, H, N, T, Z, FF), POM 3(C, I, O, U, AA, GG), 3F4 (D, J, P, V, BB, HH), 6H4 (E, K, Q, W, CC, II) or POM 19 (F, L, R, X, DD, JJ). The schematic at the bottom of the figure represents the location of the anti-PrP antibodies and the HY and DY PrP^Sc^ PK cleavage sites are depicted as solid and dashed lines, respectively. Scale bar, 50.
(8.23 MB TIF)

Figure S7 Processing of PrP^Sc^ in microglia is not strain specific. Dual fluorescence PrP^Sc^/IbA-1 immunohistochemistry was performed on CNS tissue of hamsters at the clinical stage of disease infected with either the 263K (A–F), HaCWD (G–L), 22AH (M–R), 22CH (S–X), 139H (Y–DD), or ME7H (EE–JJ) agents using the anti-PrP antibodies 8B4 (A, G, M, S, Y, EE), BE12 (B, H, N, T, Z, FF), POM 3(C, I, O, U, AA, GG), 3F4 (D, J, P, V, BB, HH), 6H4 (E, K, Q, W, CC, II) or POM 19 (F, L, R, X, DD, JJ). The schematic at the bottom of the figure represents the location of the anti-PrP antibodies and the HY and DY PrP^Sc^ PK cleavage sites are depicted as solid and dashed lines, respectively. Scale bar, 50.
(8.18 MB TIF)

Table S1 Semi-quantification of PrP^Sc^ deposition in neurons and glia from hamsters infected with 8 different prion strains using a panel of anti-PrP antibodies.
(0.09 MB DOC)
