# Supplementary figures and images for: The Strain-Encoded Relationship between PrPSc Replication, Stability and Processing in Neurons is Predictive of the Incubation Period of Disease
Source: PLoS Pathog. 2011 Mar 17;7(3):e1001317. doi: 10.1371/journal.ppat.1001317 (PMC3060105; doi:10.1371/journal.ppat.1001317)

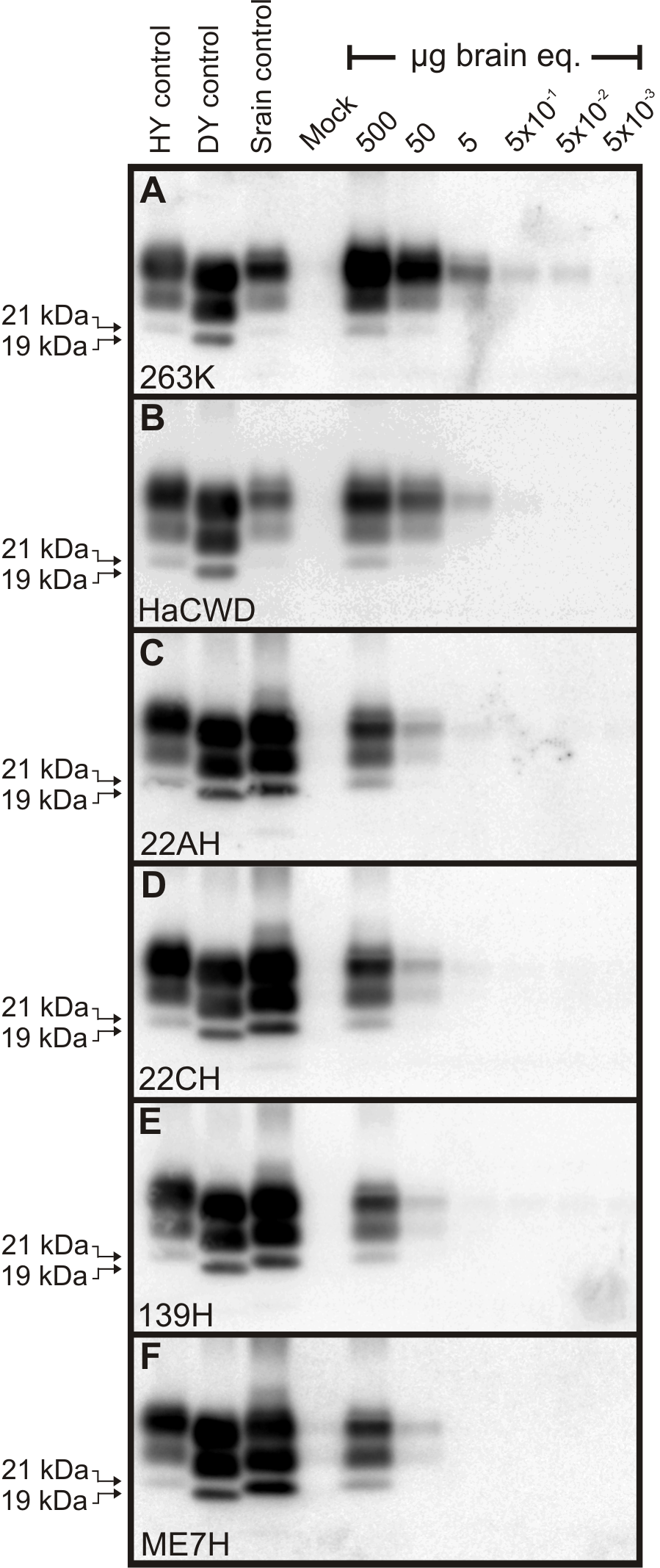

Supplement: Figure S1 — PMCA replication efficiency of hamster adapted prion strains. Western blot analysis of PrPSc following one round of PMCA that was performed on 10 fold serial dilutions of brain homogenate from hamster infected with either the (A) 263K, (B) HaCWD, (C) 22AH, (D) 22CH, (E) 139H, or (F) ME7H agents. A mock infected negative control was included in every experiment. The migration of the 19 and 21 kDa unglycosylated PrPSc polypeptides is indicated on the left of each panel. (5.76 MB TIF) [file ppat.1001317.s001.tif]

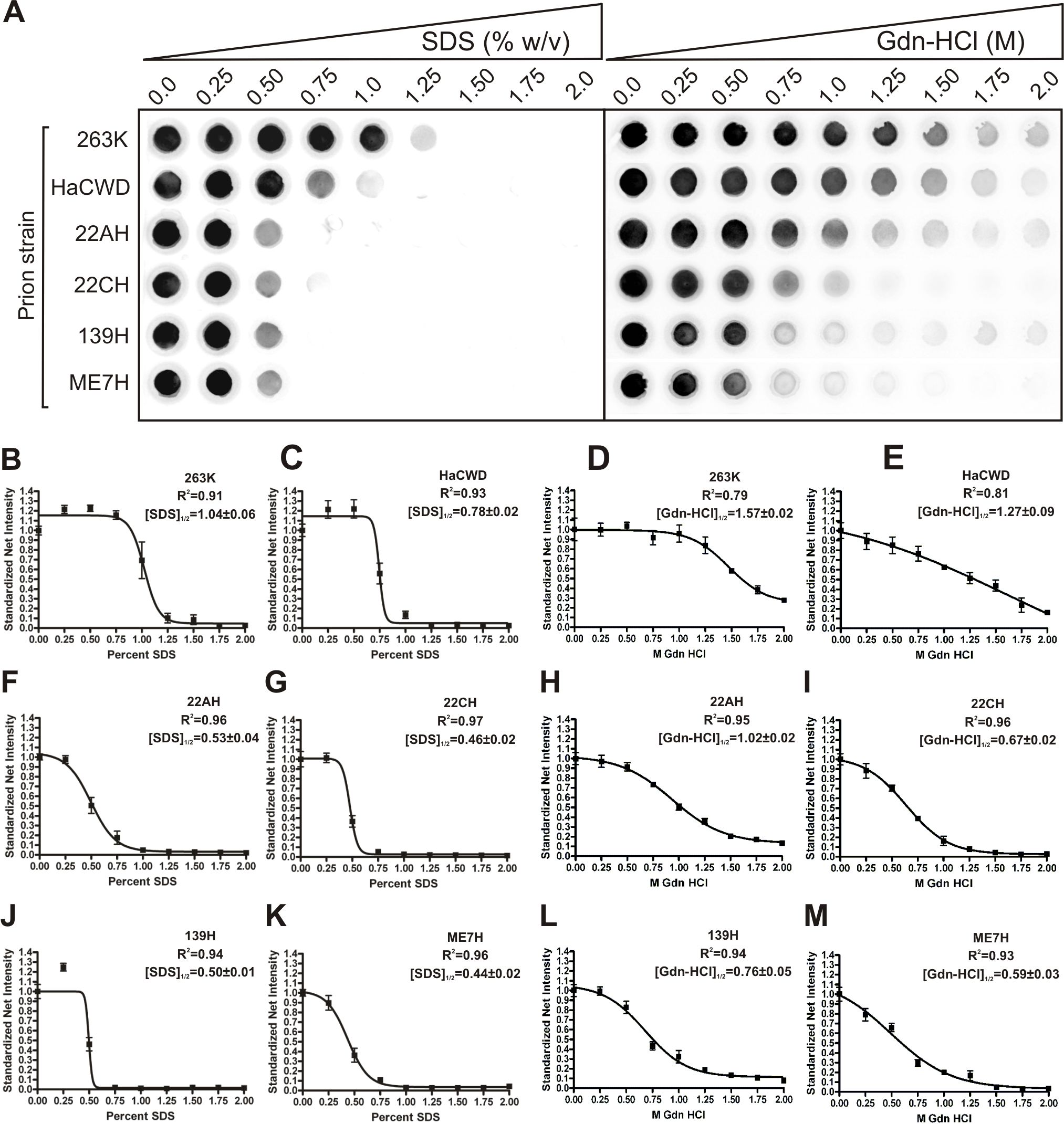

Supplement: Figure S2 — PrPSc conformational stability assays for multiple hamster-adapted prion strains. A) Brain homogenate from prion-infected hamsters were subject to incubation with increasing concentrations of either SDS or Gdn-HCl, digested with PK and the remaining PrPSc was detected using a 96-well immunoassay. The corresponding [SDS]1/2 and [Gdn-HCl]1/2 values were calculated from hamsters infected with either the (B,D) 263K, (C,E) HaCWD, (F,H) 22AH, (G,I) 22CH, (J,L) 139H, or (K,M) ME7H agents. (1.15 MB TIF) [file ppat.1001317.s002.tif]

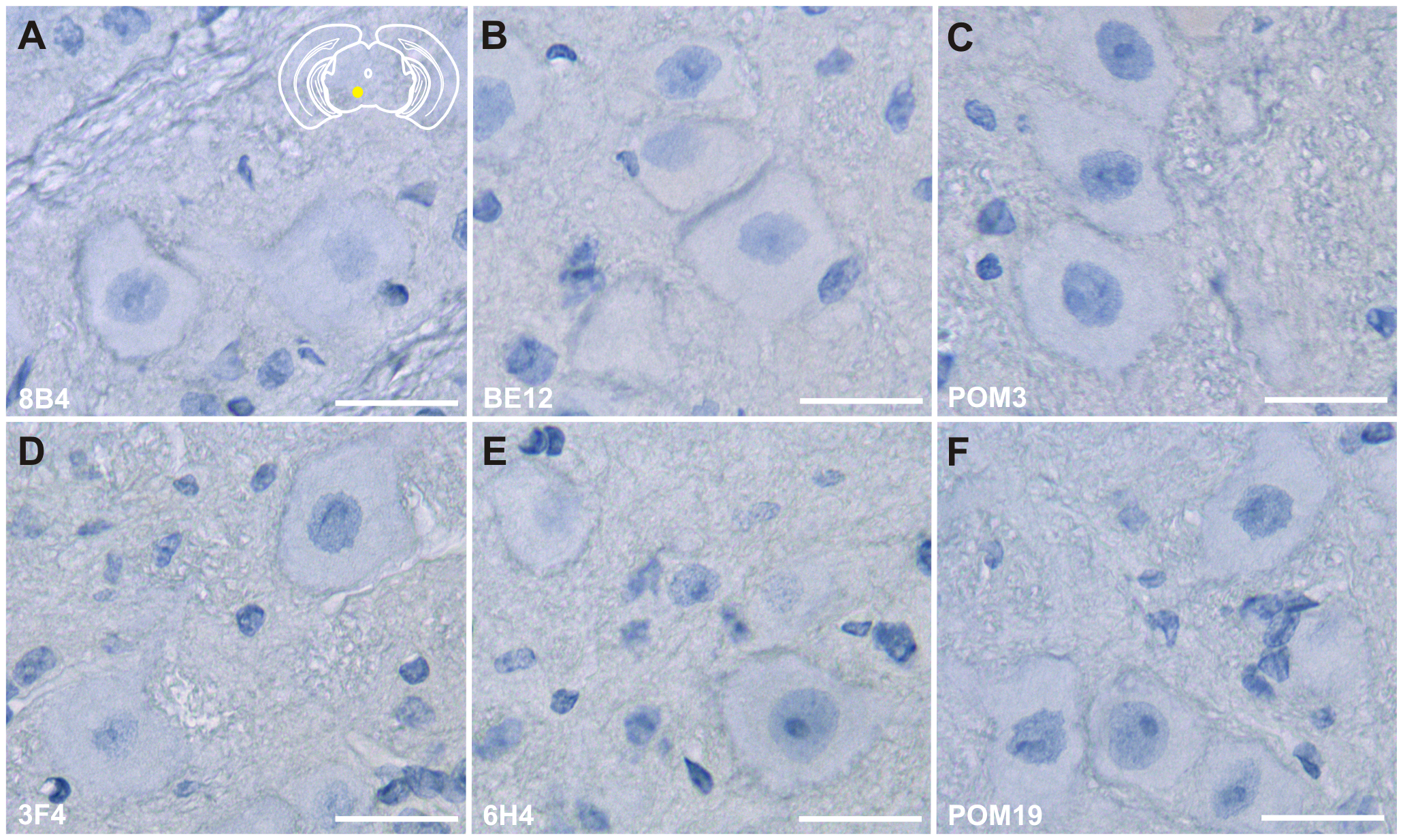

Supplement: Figure S3 — Specificity of anti-PrP antibodies for PrPSc immunodetection in the CNS of hamsters. PrPSc immunohistochemistry was performed on sections of red nucleus of a mock-inoculated animal using the anti-PrP antibodies (A) 8b4, (B) BE12, (C) POM 3, (D) 3F4, (E) 6H4, and (F) POM19 whose epitopes span from the N-terminal to C-terminal of PrP (Table 2). Scale bar, 50 µm. (7.64 MB TIF) [file ppat.1001317.s003.tif]

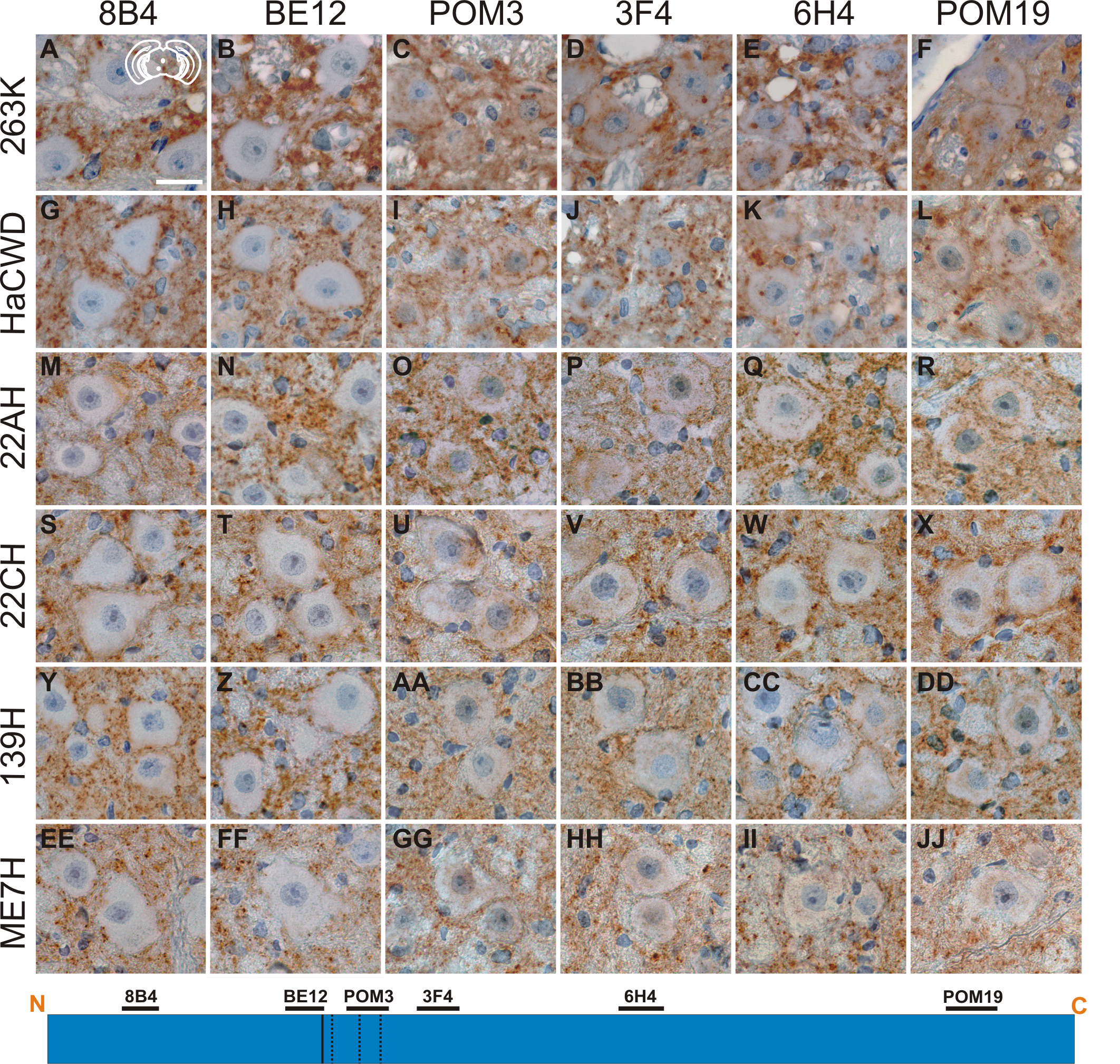

Supplement: Figure S4 — Intrasomal deposition of PrPSc in neurons is a property of short incubation period strains in hamsters. PrPSc immunohistochemistry was performed on CNS tissue of hamsters at the clinical stage of disease following infection with either the 263K (A–F), HaCWD (G–L), 22AH (M–R), 22CH (S–X), 139H (Y–DD), or ME7H (EE–JJ) agents using the anti-PrP antibodies 8B4 (A, G, M, S, Y, EE), BE12 (B, H, N, T, Z, FF), POM 3(C, I, O, U, AA, GG), 3F4 (D, J, P, V, BB, HH), 6H4 (E, K, Q, W, CC, II) or POM 19 (F, L, R, X, DD, JJ). The schematic at the bottom of the figure represents the location of the anti-PrP antibodies and the HY and DY PrPSc PK cleavage sites are depicted as solid and dashed lines, respectively. Scale bar, 50 µm. (9.06 MB TIF) [file ppat.1001317.s004.tif]

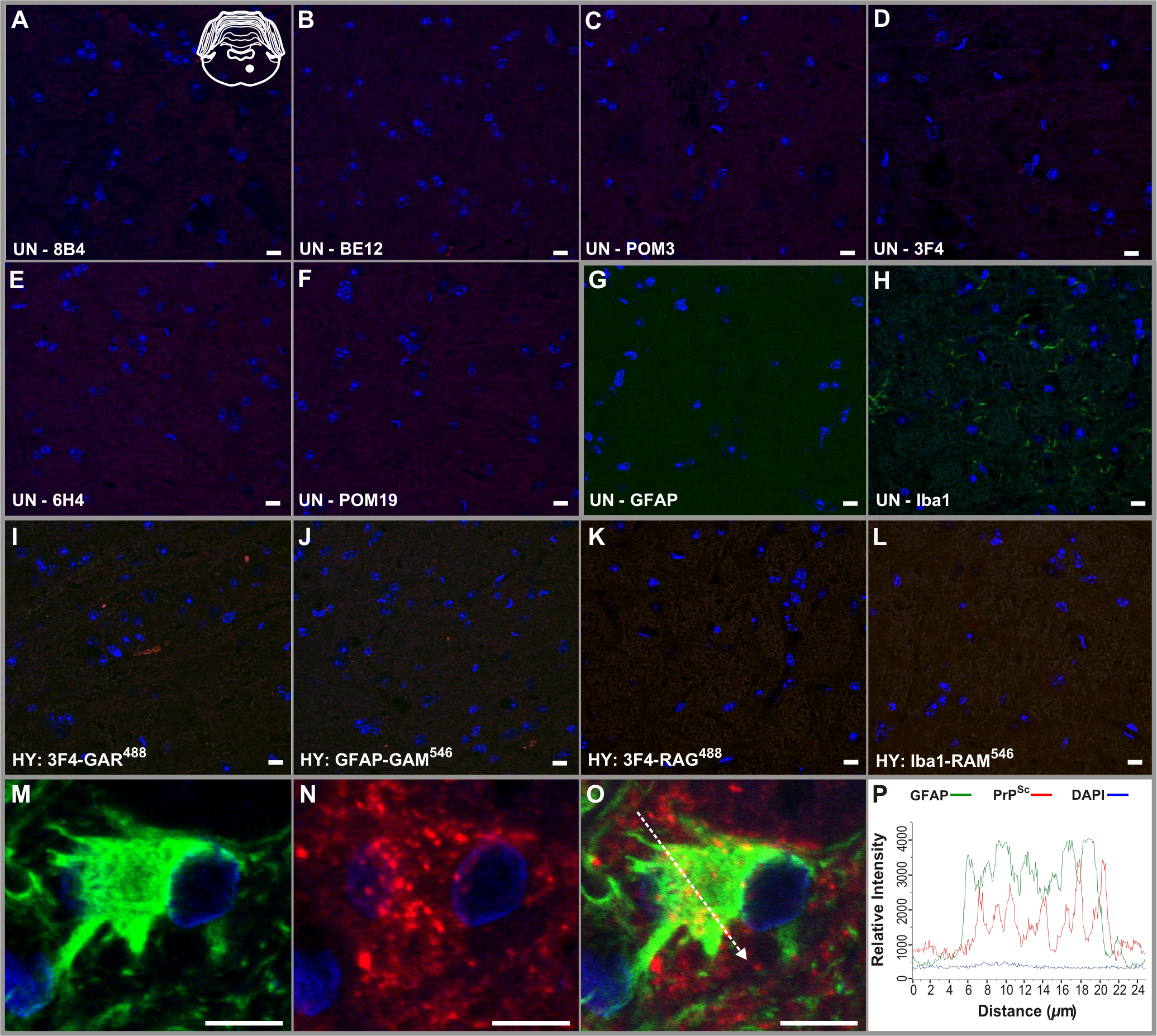

Supplement: Figure S5 — Specificity of immunolabeling and criteria of immunolabel co-localization. PrPSc immunofluorescence was performed on the reticular formation of a negative control mock-inoculated animal using the anti-PrP antibodies (A) 8b4, (B) BE12, (C) POM 3, (D) 3F4, (E) 6H4, or (F) POM19 and antibodies directed against (G) GFAP or (H) Iba-1. Non-specific binding of the monoclonal antibodies or fluorescently conjugated secondary antibodies was discounted by switching the appropriate secondary antibodies for (I, K) PrP, (J) GFAP, or (L) Iba-1. To determine co-localization of PrPSc within astrocytes or microglia using confocal microscopy, the relative fluorescence intensities of GFAP (M) and PrPSc (N) from the same 1 µm optical slice was merged (O) and a the relative intensities of the GFAP and PrPSc signals were determined along a line through the length of the cell (P). The solid white circle located in the schematic inset is the location of the photographed images within the reticular formation. Scale bar, 10 µm. (6.42 MB TIF) [file ppat.1001317.s005.tif]

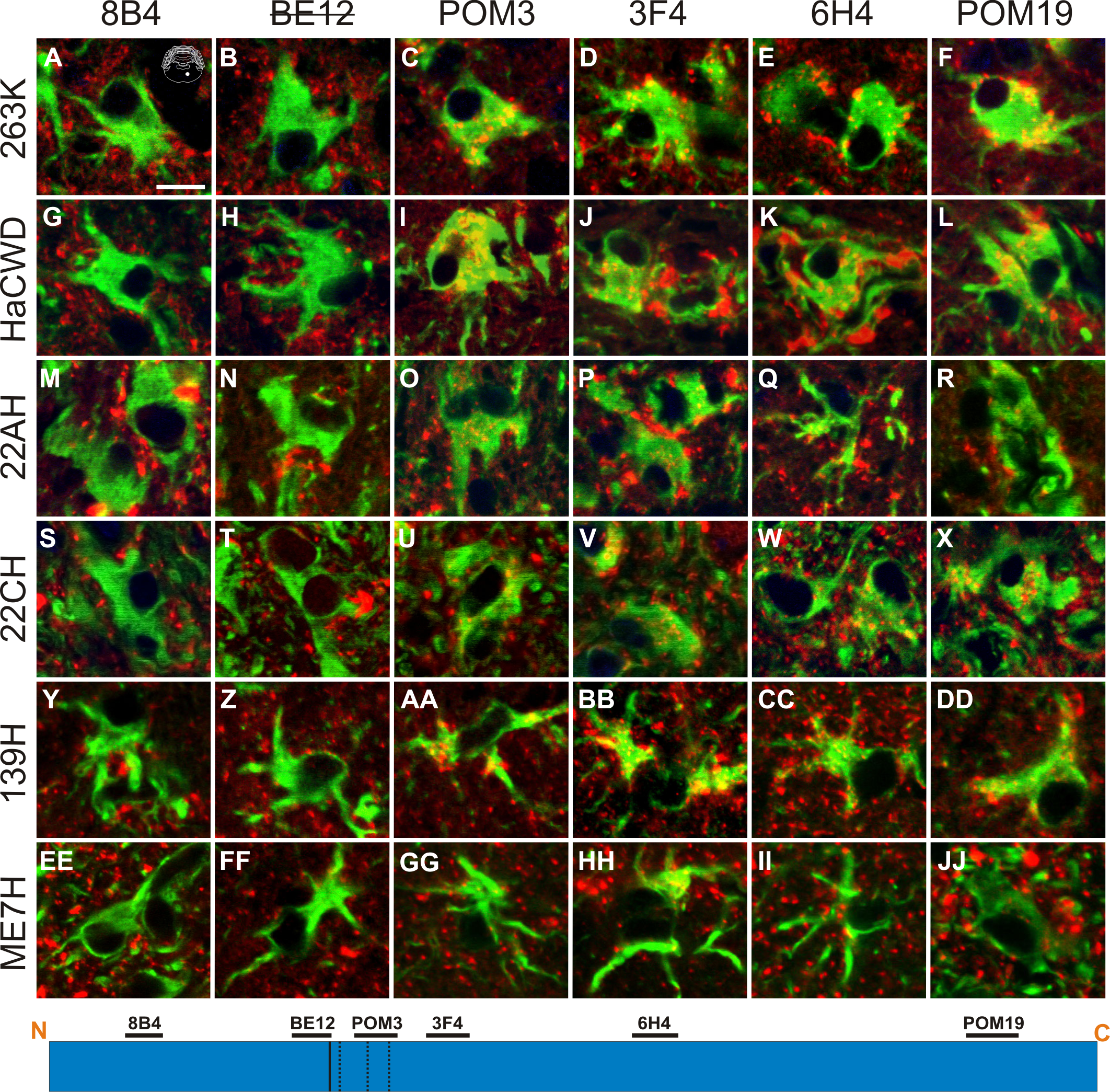

Supplement: Figure S6 — Similar N-terminal truncation of PrPSc in astrocytes of hamster-adapted strains. Dual fluorescence PrPSc/GFAP immunohistochemistry was performed on CNS tissue of hamsters at the clinical stage of disease following infection with either the 263K (A–F), HaCWD (G–L), 22AH (M–R), 22CH (S–X), 139H (Y–DD), or ME7H (EE–JJ) agents using the anti-PrP antibodies 8B4 (A, G, M, S, Y, EE), BE12 (B, H, N, T, Z, FF), POM 3(C, I, O, U, AA, GG), 3F4 (D, J, P, V, BB, HH), 6H4 (E, K, Q, W, CC, II) or POM 19 (F, L, R, X, DD, JJ). The schematic at the bottom of the figure represents the location of the anti-PrP antibodies and the HY and DY PrPSc PK cleavage sites are depicted as solid and dashed lines, respectively. Scale bar, 50. (8.23 MB TIF) [file ppat.1001317.s006.tif]

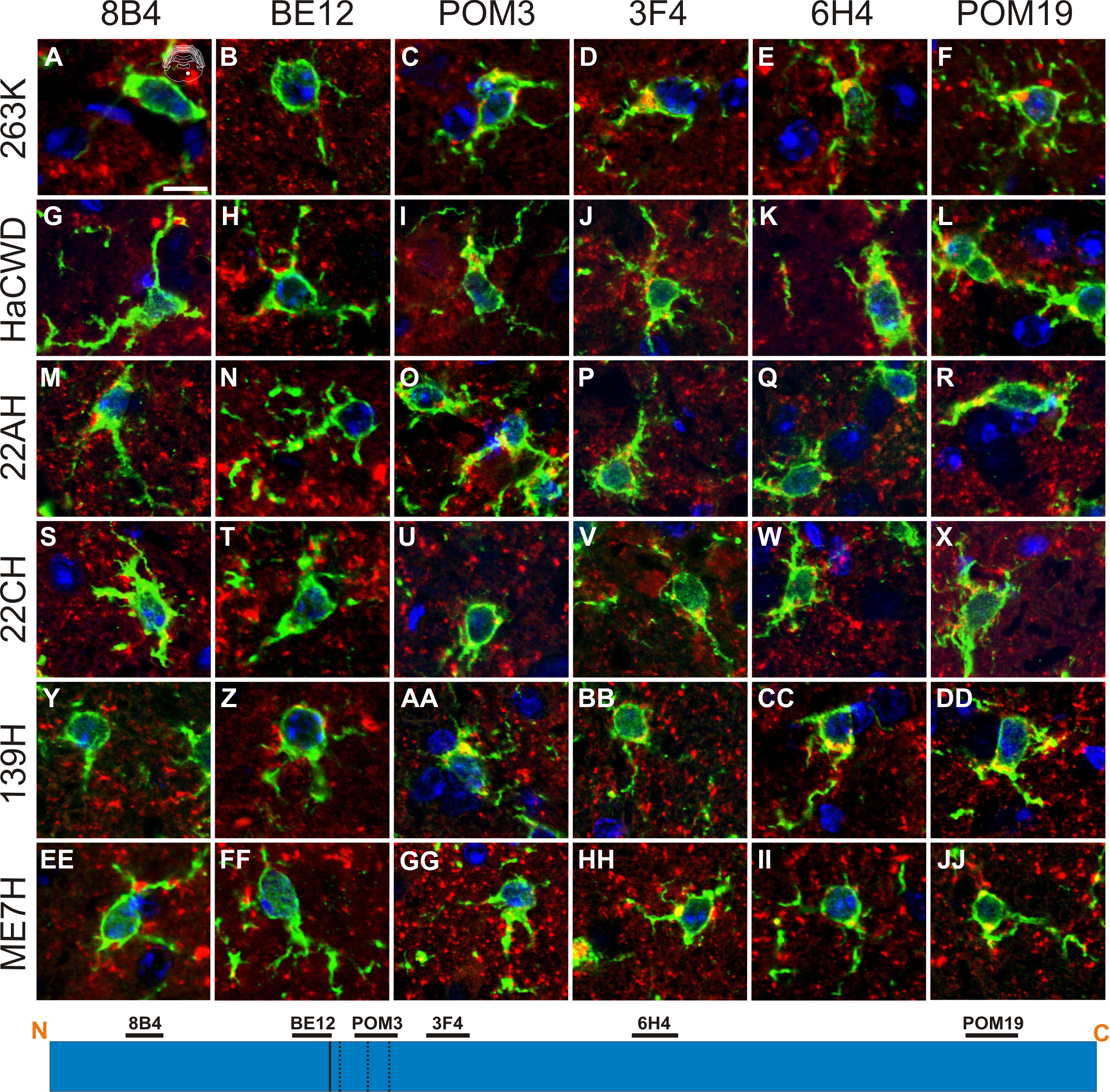

Supplement: Figure S7 — Processing of PrPSc in microglia is not strain specific. Dual fluorescence PrPSc/IbA-1 immunohistochemistry was performed on CNS tissue of hamsters at the clinical stage of disease infected with either the 263K (A–F), HaCWD (G–L), 22AH (M–R), 22CH (S–X), 139H (Y–DD), or ME7H (EE–JJ) agents using the anti-PrP antibodies 8B4 (A, G, M, S, Y, EE), BE12 (B, H, N, T, Z, FF), POM 3(C, I, O, U, AA, GG), 3F4 (D, J, P, V, BB, HH), 6H4 (E, K, Q, W, CC, II) or POM 19 (F, L, R, X, DD, JJ). The schematic at the bottom of the figure represents the location of the anti-PrP antibodies and the HY and DY PrPSc PK cleavage sites are depicted as solid and dashed lines, respectively. Scale bar, 50. (8.18 MB TIF) [file ppat.1001317.s007.tif]
