# Supplementary material for: The Strain-Encoded Relationship between PrPSc Replication, Stability and Processing in Neurons is Predictive of the Incubation Period of Disease
Source: PLoS Pathog. 2011 Mar 17;7(3):e1001317. doi: 10.1371/journal.ppat.1001317 (PMC3060105; doi:10.1371/journal.ppat.1001317)
Supplement: Table S1 — Semi-quantification of PrPSc deposition in neurons and glia from hamsters infected with 8 different prion strains using a panel of anti-PrP antibodies. (0.09 MB DOC) [file ppat.1001317.s008.doc]

**Table S1.** Semi-quantification of PrP^Sc^ deposition in neurons and glia from hamsters infected with 8 different prion strains using a panel of anti-PrP antibodies.

| Location / Strain | Anti-PrP Antibody | | | | | |
| --- | --- | --- | --- | --- | --- | --- |
|  | 8B4 | BE12 | POM3 | 3F4 | 6H4 | POM19 |
| **Neuropil** |  |  |  |  |  |  |
| HY TME | 2.89±0.11^a^ DF^b^ | 2.89±0.11 DF | 2.67±0.24 CD | 2.78±0.15 DE | 2.33±0.24 CE | 2.22±0.28 AC |
| HaCWD | 2.83±0.17 DEF | 3.00±0.00 DF | 2.50±0.22 ADE | 3.00±0.00 EF | 2.67±0.21 DE | 2.33±0.21 AE |
| 263K | 2.33±0.33 AD | 2.67±0.21 BDF | 2.00±0.37 AB | 2.33±0.33 AD | 2.67±0.33 BDE | 1.83±0.31 A |
| 22AH | 3.00±0.00 EF | 3.00±0.00 F | 2.67±0.24 DF | 3.00±0.00 EF | 3.00±0.00 DF | 3.00±0.00 F |
| 22CH | 3.00±0.00 EF | 2.89±0.11 F | 3.00±0.00 DF | 3.00±0.00 EF | 3.00±0.00 DF | 3.00±0.00 F |
| 139H | 3.00±0.00 EF | 3.00±0.00 F | 2.78±0.15 DF | 3.00±0.00 EF | 3.00±0.00 DF | 2.67±0.17 CEF |
| DY TME | 3.00±0.00 EF | 3.00±0.00 EF | 2.67±0.17 DE | 3.00±0.00 E | 2.56±0.18 DE | 2.78±0.15 EF |
| ME7H | 2.89±0.11 DEF | 2.67±0.17 EF | 2.67±0.17 DE | 2.89±0.11 DE | 2.78±0.15 DE | 2.56±0.18 CEF |
| **Intraneuronal** |  |  |  |  |  |  |
| HY TME | 0.00±0.00 A | 0.00±0.00 A | 2.11±0.35 EF | 3.00±0.00 G | 2.67±0.17 FG | 2.44±0.24 FG |
| HaCWD | 0.00±0.00 A | 0.00±0.00 A | 2.00±0.37 E | 1.67±0.21 DE | 0.83±0.31 BC | 1.17±0.17 CD |
| 263K | 0.00±0.00 A | 0.00±0.00 A | 2.17±0.31 E | 1.83±0.31 E | 1.67±0.21 E | 1.50±0.22 DE |
| 22AH | 0.00±0.00 A | 0.22±0.15 AB | 1.11±0.11 C | 0.78±0.15 BC | 0.78±0.15 BC | 0.78±0.15 BC |
| 22CH | 0.00±0.00 A | 0.00±0.00 A | 1.00±0.29 C | 0.67±0.17 C | 0.67±0.24 BC | 0.89±0.11 BCD |
| 139H | 0.11±0.11 AB | 0.00±0.00 A | 1.00±0.00 C | 0.89±0.20 C | 0.56±0.18 AC | 0.67±0.17 BC |
| DY TME | 0.44±0.18 AC | 0.11±0.11 A | 0.00±0.00 A | 1.00±0.00 CD | 0.33±0.17 AB | 0.89±0.11 BCD |
| ME7H | 0.22±0.15 AB | 0.22±0.15 AB | 0.78±0.15 BC | 1.11±0.26 CD | 0.56±0.18 AC | 0.44±0.18 AB |
| **Intra-astrocytic** |  |  |  |  |  |  |
| HY TME | 0.00±0.00 A | 0.00±0.00 A | 2.67±0.17 G | 2.83±0.15 FG | 2.67±0.17 FG | 2.67±0.17 G |
| HaCWD | 0.00±0.00 A | 0.00±0.00 A | 2.50±0.22 EG | 2.00±0.26 EH | 2.00±0.26 EF | 2.00±0.00 EG |
| 263K | 0.00±0.00 A | 0.00±0.00 A | 2.83±0.17 G | 2.33±0.21 FGH | 2.00±0.00 EF | 2.33±0.21 FG |
| 22AH | 0.22±0.15 A | 0.44±0.18 A | 1.67±0.17 D | 1.56±0.18 DE | 1.22±0.28 DH | 1.67±0.17 DEF |
| 22CH | 0.33±0.17 A | 0.56±0.18 A | 1.78±0.15 D | 1.56±0.18 DE | 1.44±0.18 DE | 1.22±0.22 CD |
| 139H | 0.22±0.15 A | 0.33±0.17 A | 1.44±0.24 D | 1.00±0.24 BD | 1.00±0.24 BD | 1.44±0.18 DE |
| DY TME | 0.00±0.00 A | 0.22±0.15 AB | 0.11±0.11 A | 1.44±0.18 DE | 1.67±0.17 EH | 0.78±0.15 BC |
| ME7H | 0.22±0.15 A | 0.33±0.17 A | 0.56±0.18 A | 0.78±0.22 AB | 0.44±0.18 AB | 0.44±0.18 AB |
| **Intra-microglial** |  |  |  |  |  |  |
| HY TME | 0.22±0.15 A | 0.44±0.18 A | 2.11±0.26 DEF | 2.33±0.24 EF | 1.44±0.24 BD | 2.11±0.20 DE |
| HaCWD | 0.50±0.22 A | 0.67±0.21 A | 2.33±0.21 EFG | 2.17±0.40 DEF | 1.83±0.31 DE | 1.33±0.21 AD |
| 263K | 1.00±0.00 AB | 0.50±0.22 A | 2.50±0.22 EF | 3.00±0.00 F | 2.33±0.21 EF | 1.67±0.33 BDE |
| 22AH | 0.44±0.18 A | 0.33±0.17 A | 2.67±0.17 FG | 2.11±0.20 DEF | 1.56±0.18 DE | 1.78±0.15 BD |
| 22CH | 0.44±0.24 A | 0.67±0.17 AB | 1.56±0.18 CD | 1.00±0.17 AC | 0.78±0.15 ABC | 1.44±0.24 BCD |
| 139H | 0.44±0.18 A | 0.56±0.18 AB | 1.67±0.29 CDE | 1.67±0.24 CDE | 1.78±0.22 DE | 1.33±0.24 BD |
| DY TME | 0.78±0.22 AB | 0.44±0.18 A | 0.78±0.15 AB | 1.44±0.24 BCD | 1.89±0.11 DE | 1.44±0.18 BD |
| ME7H | 0.56±0.24 A | 0.44±0.24 A | 1.22±0.28 AC | 0.89±0.20 AB | 0.78±0.28 AB | 1.11±0.20 AB |

^a^Mean PrP^Sc^ intensity scores ± SEM

^b^Values with no letter in common are statistically different. For each location and value of PrP^Sc^ deposition, significance can be read vertically (between prion strains) and horizontally (between antibodies).
